# Association Mapping of Seedling Resistance to Tan Spot (*Pyrenophora tritici-repentis* Race 1) in CIMMYT and South Asian Wheat Germplasm

**DOI:** 10.3389/fpls.2020.01309

**Published:** 2020-08-28

**Authors:** Rahul Madhavrao Phuke, Xinyao He, Philomin Juliana, Santosh Kumar Bishnoi, Gyanendra Pratap Singh, Muhammad Rezaul Kabir, Krishna Kanta Roy, Arun Kumar Joshi, Ravi Prakash Singh, Pawan Kumar Singh

**Affiliations:** ^1^ ICAR-Indian Agriculture Research Institute, Regional Station, Indore, India; ^2^ International Maize and Wheat Improvement Centre, Texcoco, Mexico; ^3^ ICAR- Indian Institute of Wheat and Barley Research, Karnal, India; ^4^ Bangladesh Wheat and Maize Research Institute, Dinajpur, Bangladesh; ^5^ CIMMYT-India, New Delhi, India; ^6^ Borlaug Institute for South Asia, New Delhi, India

**Keywords:** tan spot, genome wide association study, seedling resistance, *Ptr* race 1, greenhouse screening

## Abstract

Tan spot caused by *Pyrenophora tritici-repentis* (*Ptr*) is an important disease of wheat in many wheat producing areas of the world. A genome wide association study (GWAS) was conducted using 11,401 SNP markers of the Illumina Infinium 15K Bead Chip with whole genome coverage to identify genomic regions associated with resistance to tan spot in a diverse panel of 184 wheat genotypes originating from South Asia and CIMMYT. The GWAS panel was phenotyped for seedling resistance to tan spot with *Ptr* race 1 in two greenhouse experiments. Besides CIMMYT germplasm, several lines from South Asia (India, Bangladesh and Nepal) showed good degree of resistance to tan spot. Association mapping was conducted separately for individual experiments and for pooled data using mixed linear model (MLM) and Fixed and random model Circulating Probability Unification (FarmCPU) model; no significant MTAs were recorded through the MLM model, whereas FarmCPU model reported nine significant MTAs located on chromosomes 1B, 2A, 2B, 3B, 4A, 5A, 5B, 6A, and 7D. The long arms of chromosomes 5A and 5B were consistent across both environments, in which the *Vrn-A1* locus was found in identified region of chromosome 5A, and MTA at IACX9261 on 5BL appears to represent the resistance gene *tsn 1*. MTAs observed on chromosomes 1B, 2A, 2B, 3B, 4A, 6A, and 7D have not been reported previously and are likely novel.

## Introduction

Wheat is a widely grown cereal crop around the world, and it is considered as staple source of nutrition for nearly 40% of the world’s population and supplies 20% of dietary protein and food calories (Giraldo et al., 2019). The forecast for global wheat utilization has been raised by 1.5 million tonnes for year 2019/20 than in 2018/19, which is mainly due to 3.5% rise in feed use demand (FAO et al., 2019). The present global wheat production is 766 million tonnes and is expected to rise to about 840 million tonnes by 2050; this demand excluded the requirement of animal feed and the adverse impact of global climate change on wheat production (Sharma et al., 2015). Hence, it is necessary to increase the wheat production to meet its increasing demand. However, changing climatic conditions and onset of severe plant disease epidemics significantly curtail the wheat grain yield and quality (Gurung et al., 2014). About 5–14% of global wheat yield is lost each year due to diseases (Oerke, 2006). A major disease of wheat is tan spot (synonym yellow spot or yellow leaf spot) which occurs in both temperate and warmer wheat growing areas in the world (Duveiller et al., 1998). This disease is caused by the necrotrophic fungal pathogen *Pyrenophora tritici-repentis* (Died.) Dreches [anamorph *Dreschslera tritici-repentis* (Died.) Shoemaker]. The tan spot fungus was first described in 1823 (Hosford, 1982), and subsequently the disease was reported from Europe, USA, and Japan in early 1900, being considered as a saprophyte causing minor to severe spotting in wheat (Wegulo, 2011). Tan spot epidemics were first reported in the 1970s from Canada, USA, Australia, and Southern Africa (Hosford, 1971; Tekauz, 1976; Rees and Platz, 1992), and it further spread to the entire Central Asia. Tan spot pathogen infects the whole plant but is generally most prominent on leaves followed by stem and head tissues. This infection leads to reduction in photosynthetic area and eventually leads to yield reduction and quality deterioration. In severe cases, the yield losses can be beyond 50% (Wegulo, 2011). In recent years this necrotrophic disease is causing increased wheat yield losses globally, which is associated with reduced tillage practices as necrotrophic pathogens overwinters in wheat stubble (Cotuna et al., 2015)

The fungus can produce at least three host selective toxins (HSTs) known as PtrToxA, PtrToxB, and PtrToxC causing chlorotic or necrotic symptoms. The toxins produced are genetically distinct on different host genotypes, based on which the tan spot isolates can be divided into eight races. The HSTs produced by the pathogen interact with the corresponding host sensitivity genes and result in compatible interaction called as effector-triggered susceptibility (ETS) which is described as an inverse gene-for-gene model or toxin model (Friesen et al., 2007). However, identification of non-race-specific resistance QTL clearly indicates that the inverse gene-for-gene model does not fully explain all interactions that occur in the tan spot pathosystem (Faris and Friesen, 2005; Faris et al., 2012). Resistance to tan spot is qualitatively or quantitatively inherited (Faris and Friesen, 2005; Chu et al., 2008; Singh et al., 2009; Chu et al., 2010; Liu et al., 2015; Singh et al., 2019; Hu et al., 2019; Liu et al., 2020), as single dominant gene *tsn 1* on chromosome 5BL (Faris et al., 2010) confers host sensitivity to Ptr ToxA. The host Ptr ToxC sensitivity gene, *Tsc1* was mapped to the short arm of chromosome 1A (Effertz et al., 2002).

Although biparental mapping was used effectively for identification of genomic regions for tan spot resistance, the limited recombination events in biparental mapping lead to limitation of identification of closely linked markers useful for MAS due to long linkage block (Riedelsheimer et al., 2012). The GWAS approach provides better resolution for identification of closely linked markers; also, it circumvents the need to develop specific mapping populations using contrasting parents, which requires long time. GWAS has previously been used for the identification of genomic regions’ resistance to tan spot in spring wheat accessions by Gurung et al. (2014), and resistance QTLs were mapped to chromosomes 2B, 4B, and 7A. Patel et al. (2013) identified 11 QTLs located on chromosomes 1A, 1D, 2B, 2D, 6A, and 7A, and Singh et al. (2016) identified QTLs on short arm of chromosomes 1A, 1B, and 6B and long arm of chromosomes 4A, 6A, 2B, 3B, 5B, and 7B; however, all three studies used General Linear Model (GLM) procedure for association analysis, which is regarded as less stringent. The present GWAS study used a diverse panel of germplasm based on collection from CIMMYT, India, Bangladesh, and Nepal. The objective of the study was to identify genomic regions associated with seedling resistance to tan spot using 184 diverse spring wheat genotypes in controlled environmental condition using mixed- linear model (MLM) and Fixed and random model Circulating Probability Unification (FarmCPU) model to identify common genomic regions.

## Material and Methods

A panel of 184 spring wheat genotypes originating from CIMMYT-Mexico (CIM-1 to CIM-97), India (IND-1 to IND-40), Bangladesh (BGD-1 to BGD-19), and Nepal (NPL-1 to NPL-28) was used in the present study (**Supplementary Table 1**). These genotypes represent the modern elite varieties and breeding lines in the respective organization or countries. Two experiments were conducted in a greenhouse for disease assessment at seedling stage. Each experiment was conducted in completely randomized design with three replications. The experimental unit consisted of four plants per entry and four checks Erik (resistant), Glenlea (susceptible), 6B-365 (moderately susceptible), and 6B-662 (moderately resistant).

### Disease Screening

For tan spot disease screening, the isolate MexPtr1 (race 1) that produces Ptr ToxA and Ptr ToxC (Singh et al., 2009) was used. The inoculation was done as described by Singh et al. (2011), and the inoculum concentration was adjusted to 4,000 conidia/ml. The seedlings were grown under controlled environmental condition in a greenhouse with the maintenance of air temperature of 20–22/16–18°C (day/night) with 16 h photoperiod. At two leaf stage or two weeks after sowing, the seedlings were inoculated with conidial suspension of the MexPtr1 isolate until runoff using a hand sprayer. After inoculation, the seedlings were incubated for 24 h under continuous leaf wetness in a mist chamber and were then returned to the greenhouse. Seedling response was evaluated seven days of post inoculation by following the 1–5 lesion rating scale developed by Lamari and Bernier (1989).

### Genotyping

The GWAS panel was genotyped with Illumina Infinium 15 K Bead Chip by Trait Genetics GmbH, Germany. Markers with missing data points more than 10% (222 markers), or minor allele frequency less than 5% (2,707 markers), or unknown position (1,695 markers) were filtered, resulting into 11,401 markers for GWAS analysis.

### Linkage Disequilibrium, Kinship, Principal Coordinate Analysis (PCA) and Population Structure Analysis

The linkage disequilibrium parameters R^2^ among the SNP markers were calculated using Tassel 5 (http://www.maizegenetics.net), and the LD estimates as the allele frequency correlation (R^2^) between SNP markers were plotted against the physical distances. A kinship matrix and clusters among individual genotypes were calculated using all 11,401 SNP markers; the heat map was generated using classical equation from Van Randen (2008) in R program. PCA analysis was performed using SNP markers, and PC1 was plotted against PC2.

The numeric transformation of genotypic data was done using XLSTAT (2010) as per required format of the Structure 2.3.4 software (Pritchard et al., 2000). The admixture model was adjusted with burn in period length for the 100,000 followed by 500,000 markers chain Monte Carlo (MCMC) replications. The subpopulation test range was kept from K1 to K10, each with five interactions (runs). The Δ K approach was used to access the actual subpopulations (Earl, 2012). Δ K was confirmed by the Evanno et al. (2005) method using the STRUCTUREHARVERSTER program (Earl, 2012). Average logarithm of the probability of the observed likelihood [LnP(D)] was calculated along with the standard deviation from the output summary. LnP(D) for each step of the MCMC was calculated for each class (K= 1 to 10) by computing the log likelihood for the data.

### Statistical and GWAS Analysis

The combined analysis of variance was carried out for the two experiments; three variance components genotypic variance $\sigma_{g}^{2}$, experimental variance $\sigma_{e}^{2}$ and interaction of genotype and experiment variance $\sigma_{g*e}^{2}$, were estimated for tan spot using restricted maximum likelihood (Patterson and Thompson, 1971) estimation procedure of GenStat software, 17^th^ edition (VSN, International, Hemel Hempstead, UK). Broad-sense-heritability was estimated using the formula:

$$H^{2}=\frac{\sigma_{g}^{2}}{\sigma_{g}^{2}+\frac{\sigma_{\epsilon }^{2}}{nrps}}$$

Where $\sigma_{g}^{2}$  presents the genetic variance, $\sigma_{\epsilon }^{2}$ represents the error variance, and *nreps* represents the number of replications.

Bartlet test was used to assess the homogeneity of error variance prior to pooling the two-experiment data for GWAS analysis. Marker-trait association (MTA) was performed using mixed- linear model (MLM) and fixed and random model circulating probability unification (FarmCPU). For GWAS analysis using MLM model, a Q + K model that considers both Kinship (K matrix) and population structure was adopted in Tassel (http://www.maizegenetics.net), whereas the FarmCPU model was performed using the R software package GAPIT v. 3.5. GWAS study was conducted for the two experiments separately as well as for the pooled experimental data. The markers were declared to be significant using Bonferroni correction with significant cutoff (p-values, 4.4 E-06) calculated at the alpha level of 0.05 using 11,401 markers to reduce false discovery rate in both MLM and FarmCPU models.

## Results

### Evaluation of Tan Spot Resistance

The coefficient of correlation between the two experiments was high, with r = 0.73 at p ≤ 0.001. The broad-sense-heritability estimate based on seedling tan spot data was 85% for experiment 1, 78% for experiment 2, and 84% for across two environments. Analysis of variance showed that variances due to genotypes $\sigma_{g}^{2}$, experiment $\sigma_{e}^{2}$ and their interaction $\sigma_{g*e}^{2}$ were all highly significant (**Table 1**). The average tan spot scores were 1.8 and 2.0 in experiments 1 and 2. The checks Erik, 6B-662, 6B-365, and Glenlea had average tan spot scores 1.0, 2.3, 2.6, and 4.6, respectively over experiments, which confirms disease induction by *P. tritici-repentis* race 1. In pooled analysis two genotypes, HD 2733 from India and BL 4407 from Nepal, were found to be highly resistant and stood above the resistant check Erik. Another 141 genotypes were found to be moderately resistant with disease scores lower than the moderately resistant check 3B-662. Twenty-one genotypes were found to be moderately susceptible in relation to checks 3B-365 and Glenlea. The top resistant lines across the two experiments included IND-17, NPL-10, CIM-87, BGD-5, CIM-50, CIM-63, IND-1, IND-27, CIM-38, CIM-55, IND-33, CIM-25, and CIM-77. The Bartlett test found that the error variances were not significantly different for the two experiments as p = 0.375 > 0.01 at Bartlett’s chi-squared (χ^2^) value with 1 degree of freedom; hence the data of the two experiments was pooled for GWAS analysis.

Table 1Analysis of variance for tan spot in the GWAS panel and distribution of tan spot score in checks and 184 wheat genotypes.

**Table:** 

Source	Df	MSS	F pro
Genotype	183	1.7253	<0.001
Experiment	1	10.522	<0.001
Genotype × Experiment	183	0.3287	<0.001
Error	721	0.1506	–

**Table:** 

Environment	Checks		GWAS panel		
	**Erik (R)**	**Glenlea (S)**	**Min**	**Max**	**Mean**
**Experiment 1**	**1.0**	**4.9**	**1.0**	**4.3**	**1.8**
**Experiment 2**	**1.1**	**4.3**	**1.0**	**3.4**	**2.0**
**Pooled**	**1.0**	**4.6**	**1.0**	**3.6**	**1.9**

### SNP Distribution for Wheat Genome, Population Structure, Kinship, PCA, and Linkage Disequilibrium Analysis

**Figure 1:**
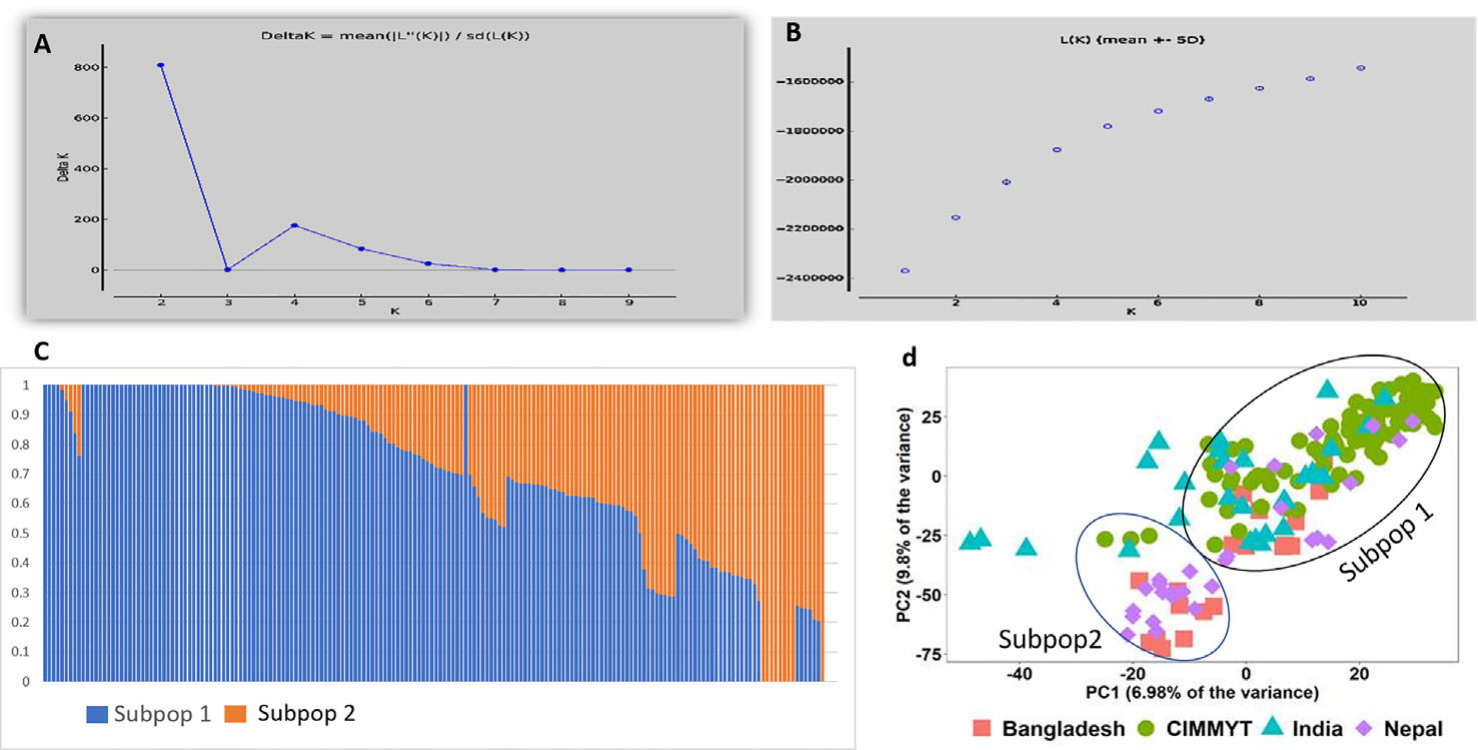
Population structure and PCA of the 184 genotypes **(A)** indicating plot of Δ k value = 2 as compared with average k =10 **(B)** Log likelihood LnP(D) *versus* the number of K. **(C)** Bar plot indicating membership coefficient (Q value at Y axis). **(D)** PCA plot showing diversity in 184 wheat genotypes.

**Figure 2:**
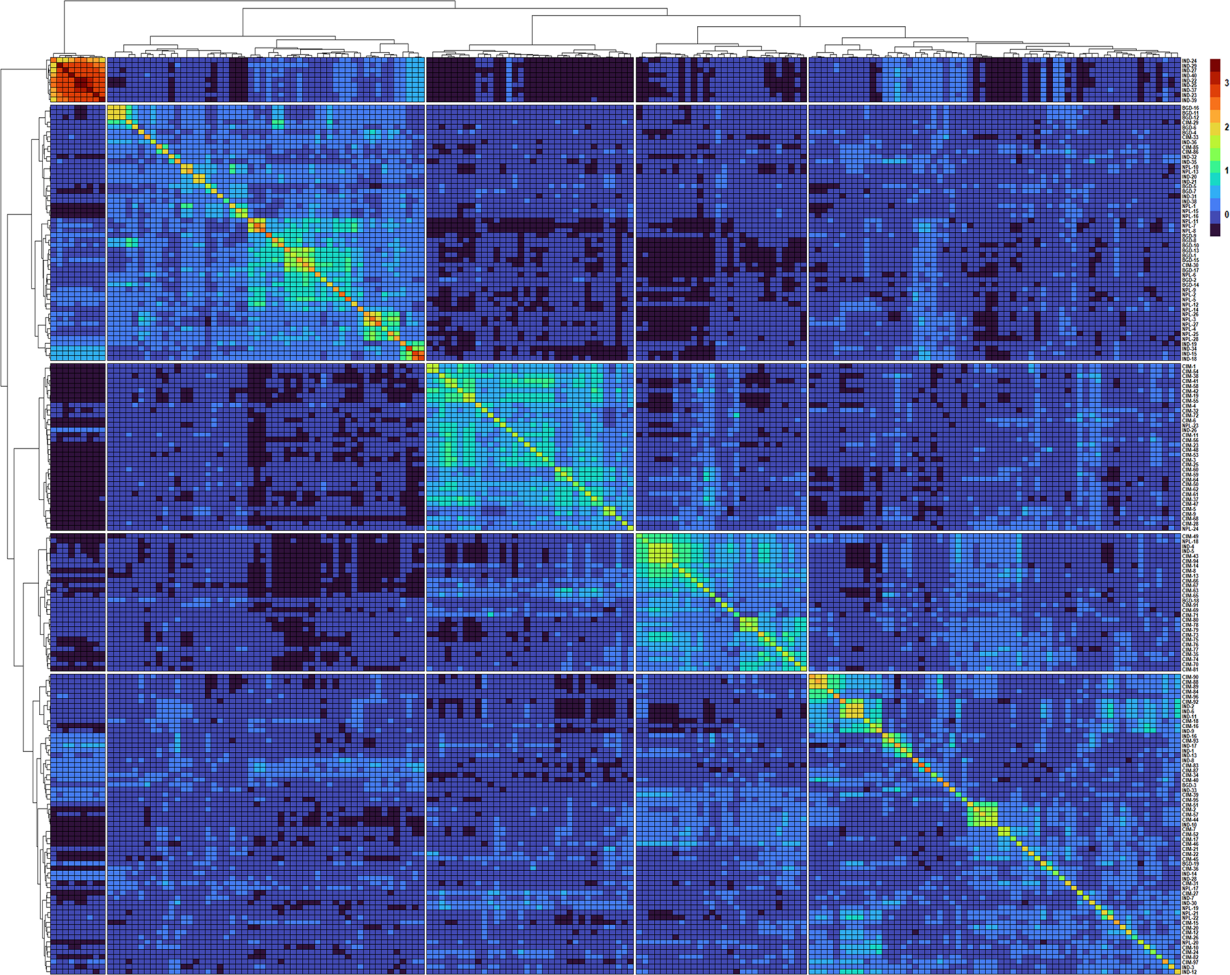
Heatmap and dendrogram of Kinship matrix estmated using Van Randen algorithum based on 11,401 SNP markers and 184 wheat genotypes.

In total 11,401 SNP markers were selected for GWAS, of which 58.5% were from the A genome, 33.9% from the B genome, and only 7.5% from the D genome. Among the 21 chromosomes, a maximum number of markers were located on chromosome 2A (1,274 markers) followed by chromosome 1A (1,119 markers), whereas the lowest number of markers was located on chromosome 4D (63 markers). Population structure analysis based on Bayesian clustering approach reveals the presence of two subpopulation (**Figures 1A–C**) and the Kinship analysis (**Figure 2**) and PCA analysis (**Figure 1D**) also divided the population into two major groups. The two sub-populations were designated as Subpop 1 and Subpop 2, which comprised 140 and 44 genotypes respectively. Subpop 1 was mainly composed of genotypes originated from CIMMYT (93 accessions), Indian breeding programs (27), with the remaining 11 lines from Nepal and 10 genotypes from Bangladesh. Most of the Indian genotypes in Subpop1, *viz.*, IND-5, IND-10, IND-3, IND-12, IND-28, IND-33, IND-9, and IND-17, were selection from the CIMMYT breeding program. Subpop 2 was made up of genotypes from Nepal (17 accessions), India (14), Bangladesh (10), and CIMMYT (3).

A clear clustering of CIMMYT and non-CIMMYT lines was also observed using Kinship analysis (**Figure 2**) and PCA analysis (**Figure 1D**). Broadly, three different groups were observed. A small group of non-CIMMYT lines includes genotypes from India, while the other two large groups which included CIMMYT and CIMMYT derived lines from India such as IND-5, IND-10, IND-3, IND-12, IND-28, IND-33, IND-9, and IND-17 and also a few from Bangladesh. In general, CIMMYT lines with common parents in pedigree cluster together, and lines that do not have common parents grouped in other clusters. Lines involving parents Super 152 (CIM-1, CIM-19, CIM-37, CIM-54, CIM-55, CIM-67, CIM-68) FRANCOLIN (CIM-14, CIM-25, CIM-41, CIM-58) and BAJ (CIM-5, CIM-9, CIM-28, CIM-37, CIM-59, CIM-60, CIM-61, CIM-62) distinguished themselves by clustering together. Likewise, lines with Saual (CIM-70, CIM-78, CIM-79, CIM-80, CIM-81), Kachu (CIM-43, CIM-49, CIM-63, CIM-66, CIM-73, CIM-74, CIM-75, CIM- 76, CIM- 77, CIM-94), Attila (CIM-65), and PBW 65 (CIM-69, CIM-71) parents were clustered together. Also, sib from BORL 14 (CIM-70, CIM-77, CIM-78, CIM-79) and KFA (CIM-71, CIM-73, CIM-74, CIM-75, CIM-76) and sib from FINSI (CIM-90, CIM-96) and METSO (CIM-84, CIM-88, CIM-89) were clustered separately.

**Figure 3:**
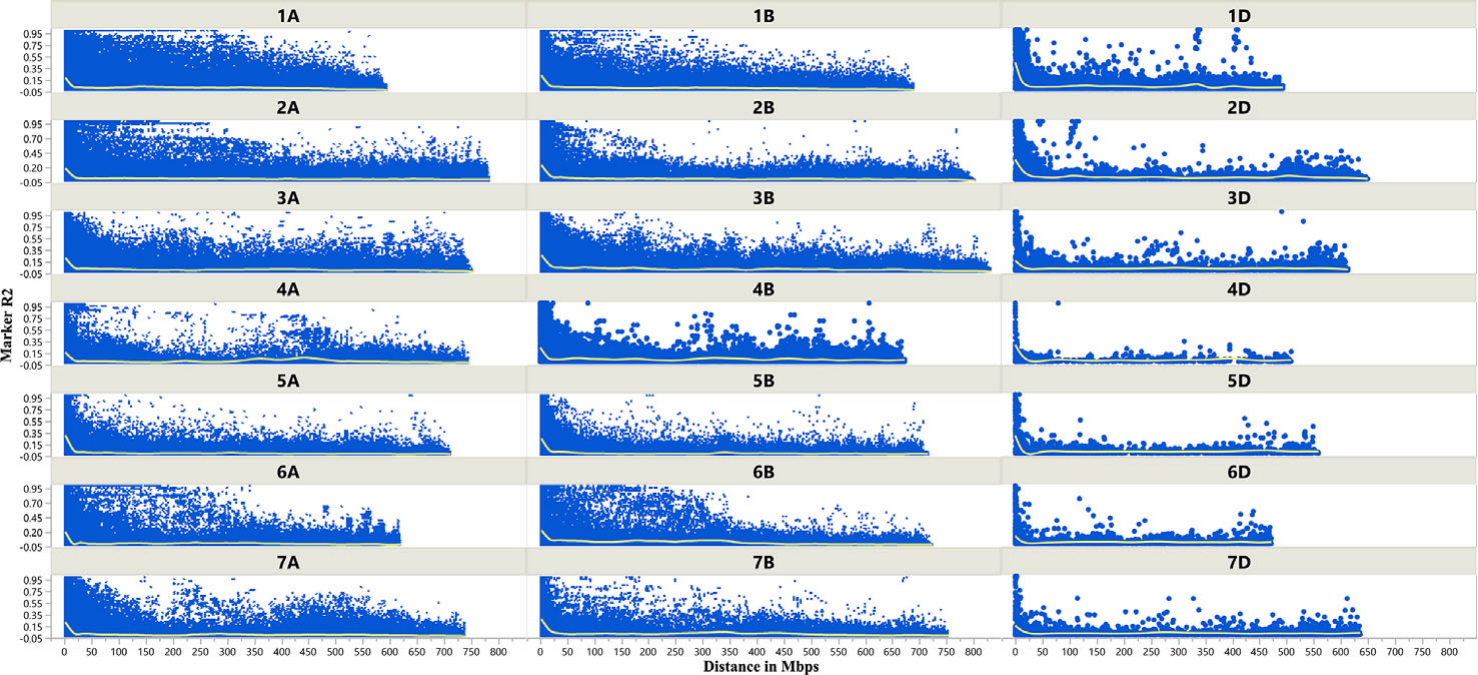
Scatter plot showing linkage disequilibrium (LD) decay across the chromosomes of wheat. Physical distance in Mb is plotted against the LD estimate (*R* ^2^) for pairs of markers associated with tan spot.

The LD decay plots (**Figure 3**) were plotted using physical distances in mega base pairs (Mb) against marker *R*2 across the chromosomes. The average extent of LD, considered as physical distance taken for decay of *R*2 to a critical value of 0.10 across the genome, was approximately 25 Mb.

### Marker Trait Association (MTA) for Tan Spot

An MLM model, a Q + K model that considers both Kinship (K matrix) and population structure, reported no significant MTAs at LOD 4.75, whereas MTAs reported at LOD 3 for MLM model were depicted in **Supplementary Figure 1**.

**Figure 4:**
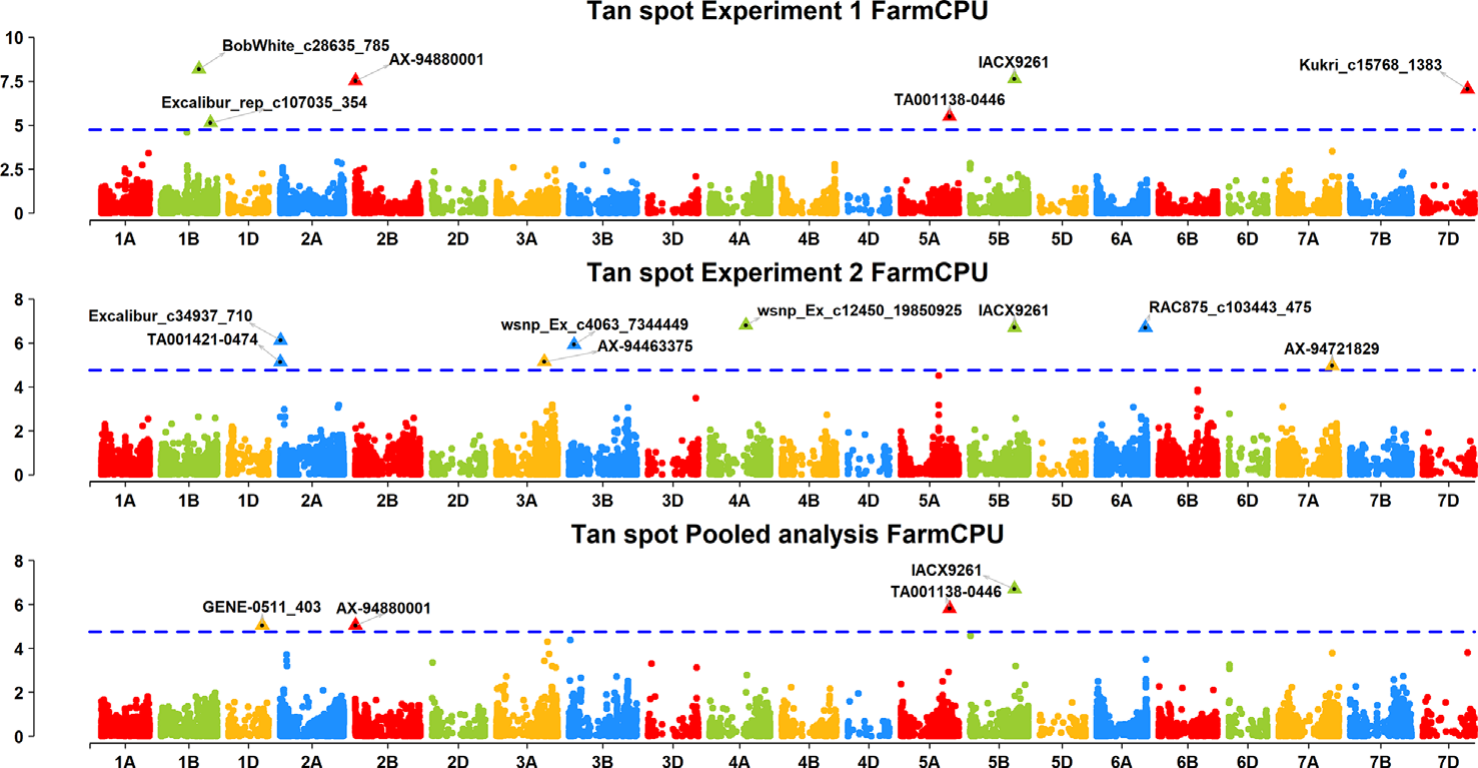
Manhattan plots based on FarmCPU model indicating associated markers and chromosome in experiment 1, experiment 2 and pooled analysis. Foot note: X axis—chromosomes, Y axis—LOD score.

**Figure 5:**
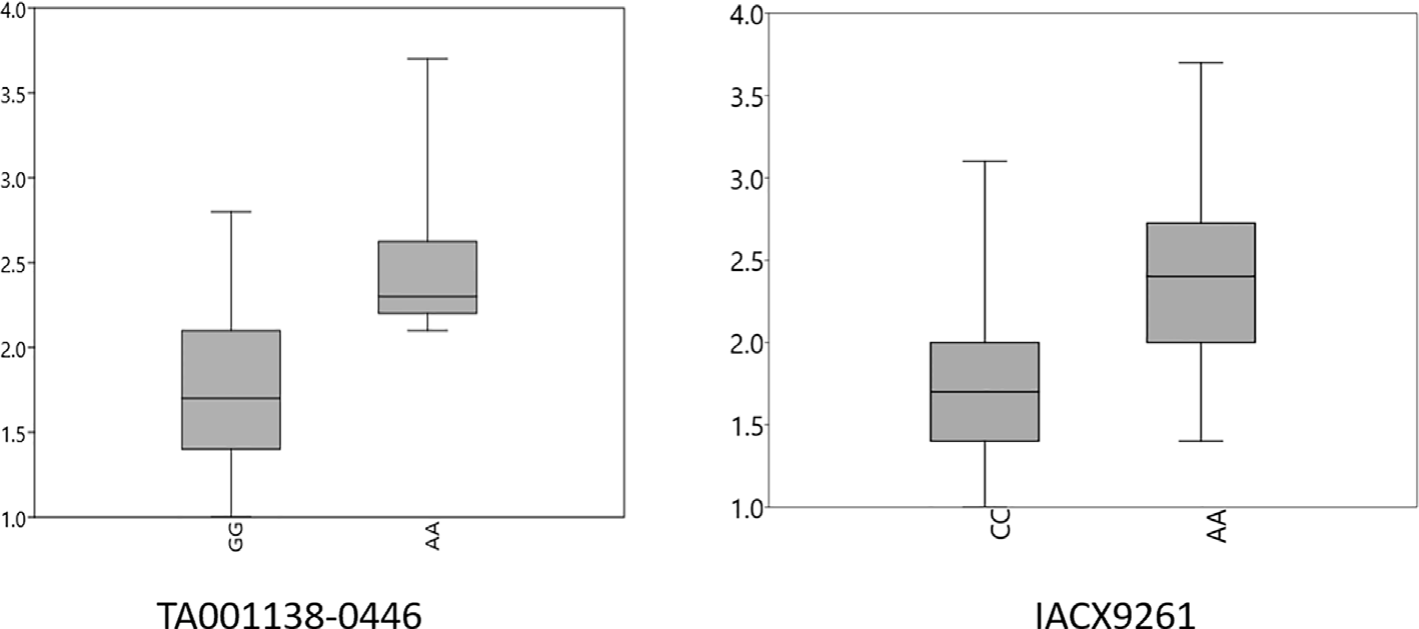
Box plots for effects of resistant and susceptible alleles on average tan spot score for stable MTAs. Foot note: X axis—resistant and susceptible allele, Y axis—average tan spot score.

A FarmCPU model, having advantage over the MLM model, has high power and less false positive through iterative usage of fixed and random effect and was also used to identify significant MTAs. Total nine MTAs showed significant association with tan spot using Bonferroni correction cutoff (p-values, 4.4 E-06) spreading over eight chromosomes *viz.*, 2A, 2B, 3B, 4A, 5A, 5B, 6A, and 7D (**Table 2**). SNP (IACX9261) on chromosome 5B was most stable and consistent in both individual experiments and in pooled analysis, followed by SNP (TA001138-0446) on chromosome 5A and SNP (AX- 94880001) on chromosome 2B which are common among experiment 1 and in pooled analysis. The remaining five MTAs are exclusive to either experiment 1 or experiment 2. The significant markers with LOD scores in the Manhattan plot are presented in **Figure 4**. The R^2^ explained by significant markers range from 2.0 to 11.3%. The highest R^2^ (11.3) was explained by SNP (IACX9261) on chromosome 5B. Comparison of significant MTAs identified from individual environment and pooled analysis showed that genomic regions on the long arm of chromosome 5A and 5B are most stable. The effects of resistant and susceptible alleles for MTAs on chromosomes 5A and 5B were shown with box plot in **Figure 5**.

**Table 2:** Markers significantly associated with seedling resistance to tan spot through FarmCPU model.

SNP	Chromosome	Position	P. value	marker R^2^	Experiment
BobWhite_c28635_785	1B	465584555	6.41E-09	0.060	Exp 1
Excalibur_c34937_710	2A	4789172	7.65E-07	0.031	Exp2
AX-94880001	2B	3312733	2.94E-08	0.063	Exp 1, Pooled
wsnp_Ex_c4063_7344449	3B	64175429	1.22E-06	0.012	Exp2
wsnp_Ex_c12450_19850925	4A	446471067	1.58E-07	0.060	Exp2
TA001138-0446	5A	597291565	1.57E-06	0.074	Pooled, Exp 1
IACX9261	5B	546703936	2.33E-08	0.113	Exp 1, Exp 2, Pooled
RAC875_c103443_475	6A	596903177	2.01E-07	0.040	Exp2
Kukri_c15768_1383	7D	550216751	8.96E-08	0.020	Exp 1

Physical position for SNPs referred from the Chinese Spring RefSeq ver. 1.0.

The presence and absence of resistant alleles for total nine identified MTAs from both the models were examine in all 184 wheat genotypes (**Supplementary Table 2**). Almost all highly resistant genotypes in this panel showed the presence of resistance alleles for MTA on chromosome 5B (IACX9261); also lines with susceptible alleles for this MTA showed highly susceptible reaction as observed in genotypes IND-31, CIM-89, and CIM-16. Other MTA on 5A chromosome (**TA001138-0446**) was also found to be useful for differentiation of resistant and susceptible genotypes. Whereas other MTAs on chromosome 2B (AX-94880001), 4A (wsnp_Ex_c12450_19850925), 6A (RAC875_c103443_475), and on 7D (Kukri_c15768_1383) were observed to be less prominent in differentiation of resistant and susceptible genotypes.

## Discussion

In the present study, 184 diverse spring wheat genotypes were screened for seedling tan spot resistance in a greenhouse for the identification of significant MTAs. Field screening of large number of genotypes for tan spot is considered challenging due to the often-natural incidence of other foliar diseases that mimic tan spot symptoms; in addition, limitation of required light and humidity for inoculum growth in field condition precludes pathogen growth (Singh et al., 2009). Moreover, high level of positive correlation between greenhouse and field experiments for tan spot was observed by Evans et al. (1999), implying that MTAs from seedling experiments could be found in field experiments as well. The present study identified broad genetic base of resistance for tan spot, which includes genotypes from CIMMYT-Mexico, as well as from the three South Asian countries. The high resistance of CIMMYT germplasm was previously reported by Singh et al. (2016) and Ali et al. (2008); however, the present study adds more information using diverse genotypes other than germplasm set, which include stable breeding lines form CIMMYT international nurseries *viz.*, 40IBWSN, 28 ESWYT, and 18 HRWSN along with South Asian wheat genotypes and observed broad genetic base for resistance. The lines HD 2733 and BL 4407, newly found as highly resistant genotypes, can be used in breeding to incorporate tan spot resistance.

Prior to GWAS analysis, information about population structure is very important because the presence of population structure in the GWAS panel may cause spurious association results (Oraguzie et al., 2007). The presence of a subpopulation in a large population can be justified by selection and genetic drift (Buckler and Thornsberry, 2002). Population structure, kinship matrix, cluster analysis, and PCA analysis revealed there was moderate population structure, which has resulted from lines with common parents and two to three sibs in the pedigree.

In the present study, association analysis using MLM and FarmCPU model was adopted; the MLM model has limitation of false negative due to confounding between population structure, Kinship, and quantitative trait nucleotides; however, this limitation was overcome by using FarmCPU model as it performed marker test using associated markers as covariates in fixed effect and followed optimization with associated covariate markers in random effect, which enables to remove confounding and also control false positive (Liu et al., 2016), which is also proven from the results of quantile-quantile (QQ) plots (**Supplementary Figure 2**) which showed FarmCPU model fitted data well compared to the MLM model. Genomic regions identified for tan spot are categorized into two groups as stable and unstable. Unstable genomic regions are those which are expressed only in one experiment, whereas stable genomic regions for tan spot include the chromosomal regions which are constantly expressed across both individual experiments and pooled analysis or common either in one environment or in pooled analysis. Therefore, the genomic region on the long arm of chromosomes 5B and 5A is designated as stable and is explained here. GWAS results showed major role of *tsn 1* gene on chromosome 5B in resistance to *Ptr* race 1, as the MTA at IACX9261 that is close to *tsn 1* was stably significant across both experiments. Previous mapping studies also identified major roles of *tsn 1* in conferring resistant to *Ptr* race 1, such as Chu et al. (2008) in a doubled haploid population, Singh et al. (2008) and Faris et al. (2012) in RIL populations, Kollers et al. (2014) in European winter wheat varieties, and Liu et al., 2020 from three hexaploid wheat mapping population LP573, SK, and TN. Previously, PCR based markers *Xfcp620* and *Xfcp623* were extensively used to detect *tsn 1* gene (Lu et al., 2006; Faris et al., 2010). But from now on, the SNP IACX9261 found in the present study could also be used since it can be transformed to high throughput markers like KASP.

In addition to 5BL, another genomic region on chromosome 5AL appeared to be stable for tan spot resistance. The identified 5AL chromosome region in Chinese Spring RefSeq v. 1.0, harbors or overlaps with QTL identified in previous studies. *QTs-Fcu-5AL* flanked by markers *barc1061* and *cfd2185* by Chu et al. (2008), *QYls.lrc-5A* closely linked by *gdm132* (Zwart et al., 2010), and for *QTs.zhl-5A* mapped between markers *iwa7025* and *iwa5173* (Kariyawasam et al., 2016) were previously identified on the same genomic region. Interestingly, MTAs on chromosome 5AL (TA001138-0446 and BS00022071_51) showed tight linkage with *Vrn-A1*, which matches with our previous results (Hu et al., 2019). The *Vrn-A1* locus was reported to contribute to disease escape *via* its effects in alteration of flowering date in Fusarium head blight (He et al., 2016), spot blotch (Singh et al., 2018), and Septoria tritici blotch (Dreisigacker et al., 2015) in field condition. However, association of *Vrn-A1* with seedling resistance to tan spot and its association with spot blotch resistance even after excluding the effect of days to flowering (Singh et al., 2018) implies its possible linkage with an unknown disease resistance gene or its pleiotropic role in resistance to tan spot (Hu et al., 2019). In CIMMYT spring wheat genotypes, the *vrn-A1* allele for late flowering and tan spot resistance is almost fixed (Dreisigacker et al., 2016), which is supportive of tan spot resistance and may have contributed to a good level of tan spot resistance in CIMMYT germplasm.

Two resistance genes, *tsn 2* controlling resistance to necrosis caused by *Ptr* race 3 (Singh et al., 2006) and *tsn 5* controlling resistance to *Ptr* race 5 were reported previously in a marker interval of *gwm 285* and *wmc 366* on chromosome 3B. In the present study, we observed one significant MTA from experiment 2 on chromosome 3B, but this MTA does not match the interval between *gwm 285* and *wmc 366*. Hence it appears that this MTA does not represent *tsn 2* or *tsn 5* genes, which is in agreement with the fact that the *Ptr* isolate used in this study was for race 1 only. The significant MTAs identified on chromosomes 1B, 2A, 2B, 3B, 4A, 6A, and 7D do not match with previously identified QTL, and hence these might be novel genomic regions for resistance to *Ptr* race 1, for which further validation is needed. The single MAT on 5B (IACX9261) will be the first choice for the selection of resistant genotypes for tan spot, and in novel identified genomic regions, single MTA on chromosome 1B (BobWhite_c28635_785) will be a priority for validation as it showed better differentiation for resistant and susceptible genotypes. Overall, in this study, along with CIMMYT germplasm, diverse sources of resistant genotypes against *Ptr* race 1 were identified which can be used to develop broad genetic resistance to tan spot of wheat. Association mapping identified both known and novel QTLs for tan spot resistance along with novel markers potentially useful for marker-assisted selection. Together the identified novel resistant genotypes and genomic regions could be useful for developing cultivars with durable resistance to tan spot in wheat.

## Data Availability Statement

The datasets presented in this study can be found in online repositories. The names of the repository/repositories and accession number(s) can be found in the article/supplementary material.

## Author Contributions

PS and XH designed the research and supervised the experiments. RP and SB did the disease screening. GS, AJ, RS, MK, and KR provided plant materials. XH and PJ contributed to genotyping work. RP, XH, and PJ analyzed the data. All authors contributed to the article and approved the submitted version.

## Funding

This research was funded through several projects funded by the Indian Council of Agricultural Research, India and CGIAR Research Program on WHEAT.

## Conflict of Interest

The authors declare that the research was conducted in the absence of any commercial or financial relationships that could be construed as a potential conflict of interest.

## Supplementary Material

Supplementary Figure 1 Manhattan plots based on MLM model indicating associated markers and chromosome in experiment 1, experiment 2, and pooled analysis at LOD score 3. Foot note: X axis—chromosomes, Y axis—LOD score.

Supplementary Figure 2 Q–Q plot of observed *vs* expected log10 P values for MLM and FarmCPU GWAS models using three data sets.

Supplementary Table 1 Pedigree details of 184 spring wheat genotypes used for GWAS of Tan spot

Supplementary Table 2 Presence and absence scoring of resistant allele for identifying nine MTAs in 184 wheat genotypes

## References

[B1] Ali S. Singh P. K. McMullen M. P. Mergoum M. Adhikari T. B. (2008). Resistance to multiple leaf spotting diseases in wheat germplasm. Euphytica 159, 167–179. 10.1007/s10681-007-9469-4

[B2] Buckler E. S. I. V. Thornsberry J. M. (2002). Plant molecular diversity and applications to genomics. Curr. Opin. Plant Biol. 5, 107–111. CrossRefPubMedGoogle Scholar. 10.1016/S1369-5266(02)00238-8 11856604

[B3] Chu C. G. Friesen T. L. Xu S. S. Faris J. D. (2008). Identification of novel tan spot resistance loci beyond the known host-selective toxin insensitivity genes in wheat. Theor. Appl. Genet. 117, 873–881. 10.1007/s00122-008-0826-z 18575834

[B4] Chu C. G. Chao S. Friesen T. L. Faris J. D. Zhong S. Xu S. S. (2010). Identification of novel tan spot resistance QTLs using an SSR-based linkage map of tetraploid wheat. Mol. Breed. 25, 327–338. 10.1007/s11032-009-9335-2

[B5] Cotuna O. Paraschivu M. Paraschivu A. Saraţeanu V. (2015). The influence of tillage, crop rotation and residue management on tan spot *Drechslera tritici repentis*. Died. Shoemaker in winter wheat. Res. J. Agric. Sci. 47, 13–21.

[B6] Dreisigacker S. Wang X. Martinez C. B. A. Jing R. Singh P. K. (2015). Adult-plant resistance to Septoria tritici blotch in hexaploid spring wheat. Theor. Appl. Genet. 128, 2317. 10.1007/s00122-015-2587-9 26298303

[B7] Dreisigacker S. Sukumaran S. Guzman C. He X. Lan C. Bonnett D. (2016). “Molecular marker-based selection tools in spring bread wheat improvement: CIMMYT experience and prospects,” in Molecular Breeding for Sustainable Crop Improvement, Sustainable Development and Biodiversity, vol. Vol. 11 . Eds. Rajpal V. Rao S. Raina S. (Cham, Switzerland: Springer), 421–474.

[B8] Duveiller E. Dubin H. J. Reeves J. McNab A. (1998). Helminthosporium Blights of Wheat: Spot Blotch and Tan Spot (El Batan, Mexico: CIMMYT).

[B9] Earl D. A. (2012). STRUCTURE HARVESTER: A website and program for visualizing STRUCTURE output and implementing the Evanno method. Conserv. Genet. Resour. 4, 359–361. 10.1007/s12686-011-9548-7

[B10] Effertz R. J. Meinhardt S. W. Anderson J. A. Jordahl J. G. Francl L. J. (2002). Identification of a chlorosis-inducing toxin from *Pyrenophora tritici-repentis* and the chromosomal location of an insensitivity locus in wheat. Phytopathology 92, 527–533. 10.1094/PHYTO.2002.92.5.527 18943027

[B11] Evanno G. Regnaut S. Goudet J. (2005). Blackwell Publishing, Ltd. Detecting the number of clusters of individuals using the software STRUCTURE: a simulation study. Mol. Ecol. 14, 2611–2620. 10.1111/j.1365-294X.2005.02553.x 15969739

[B12] Evans C. K. Hunger R. M. Siegerist W. C. (1999). Comparison of greenhouse and field testing to identify wheat resistant to tan spot. Plant Dis. 83, 269–273. 10.1094/PDIS.1999.83.3.269 30845506

[B13] FAO IFAD UNICEF WFP WHO (2019). The State of Food Security and Nutrition in the World 2019. Safeguarding against economic slowdowns and downturns (Rome: FAO). Licence: CC BY-NC-SA 3.0 IGO.

[B14] Faris J. D. Friesen T. L. (2005). Identification of quantitative trait loci for race-nonspecific resistance to tan spot of wheat. Theor. Appl. Genet. 111, 386–392. 10.1007/s00122-005-2033-5 15895202

[B15] Faris J. D. Zhang Z. Lu H. J. Lu S. W. Reddy L. Cloutier S. (2010). A unique wheat disease resistance-like gene governs effector-triggered susceptibility to necrotrophic pathogens. Proc. Natl. Acad. Sci. U.S.A. 107, 13544–13549. 10.1073/pnas.1004090107 20624958 PMC2922177

[B16] Faris J. D. Abeysekara N. S. McClean P. E. Xu S. S. Friesen T. L. (2012). Tan spot susceptibility governed by the *Tsn1* locus and non race-specific resistance QTL in a population derived from the wheat lines Salamouni and Katepwa. Mol. Breed. 30, 1669–1678. 10.1007/s11032-012-9750-7

[B17] Friesen T. L. Meinhardt S. W. Faris J. D. (2007). The *Stagonospora nodorum*-wheat pathosystem involves multiple proteinaceous host-selective toxins and corresponding host sensitivity genes that interact in an inverse gene-for-gene manner. Plant J. 51, 681–692. 10.1111/j.1365-313X.2007.03166.x 17573802

[B18] Giraldo P. Benavente E. Manzano-Agugliaro F. Gimenez E. (2019). Worldwide research trends on wheat and barley: A bibliometric comparative analysis. Agronomy 9 (7), 352. 10.3390/agronomy9070352

[B19] Gurung S. Mamidi S. Bonman J. M. Xiong M. Brown-Guedira G. Adhikari T. B. (2014). Genome-wide association study reveals novel quantitative trait loci associated with resistance to multiple leaf spot diseases of spring wheat. PloS One 9 (9), e108179. 10.1371/journal.pone.0108179 25268502 PMC4182470

[B20] He X. Lillemo M. Shi J. Wu J. Bjørnstad A. Belova T. (2016). QTL Characterization of Fusarium head blight resistance in CIMMYT bread wheat line Soru1. PloS One 11 (6), e0158052. 10.1371/journal.pone.0158052 27351632 PMC4924825

[B21] Hosford R. M. J. (1971). A form of *Pyrenophora trichostoma* pathogenic to wheat and other grasses. Phytopathology 61, 28–32. 10.1094/Phyto-61-28

[B22] Hosford R. M. J. (1982). “Tan spot—developing knowledge 1902–1981, virulent races and differentials, methodology, rating systems, other leaf diseases, literature,” in Tan spot of wheat and related diseases workshop. Ed. Hosford R. M. Jr (Fargo: NDSU), pp 1–pp24.

[B23] Hu W. He X. Dreisigacker S. Carolina P. Sansaloni S. Juliana P. (2019). A wheat chromosome5AL region confers seedling resistance to both tan spot and Septoria nodorum blotch in two mapping populations. Crop J. 7 (6), 809–818. 10.1016/j.cj.2019.05.004

[B24] Kariyawasam G. K. Carter A. H. Rasmussen J. B. Faris J. Xu S. S. Mergoum M. (2016). Genetic relationships between race-nonspecific and race-specific interactions in the wheat-*Pyrenophora tritici-repentis* pathosystem. Theor. Appl. Genet. 129, 897–908. 10.1007/s00122-016-2670-x 26796533

[B25] Kollers S. Rodemann B. Ling J. Korzun V. Ebmeyer E. Argillier O. (2014). Genome-wide association mapping of tan spot resistance (*Pyrenophora tritici-repentis*) in European winter wheat. Mol. Breed. 34, 363–371. 10.1007/s11032-014-0039-x

[B26] Lamari L. Bernier C. C. (1989). Evaluation of wheat lines and cultivars to tan spot (*Pyrenophora tritici-repentis*) based on lesion type. Can. J. Plant Pathol. 11, 49–56. 10.1080/07060668909501146

[B27] Liu Z. El-Basyoni I. Kariyawasam G. Zang G. Fartiz A. Hansen J. (2015). Evaluation and association mapping of resistance to tan spot and Stagonospora nodorum blotch in adopted winter wheat germplasm. Plant Dis. 99, 1333–1341. 10.1094/PDIS-11-14-1131-RE 30690997

[B28] Liu X. Huang M. Fan B. Buckler E. S. Zhang Z. (2016). Iterative Usage of Fixed and Random Effect Models for Powerful and Efficient Genome-Wide Association Studies. PloS Genet. 12 (2), e1005767. 10.1371/journal.pgen.1005767 26828793 PMC4734661

[B29] Liu Y. Salsman E. Wang R. Galagedara N. Fiedler J. Liu Z. (2020). Meta-QTL analysis of tan spot resistance in wheat. Theor. Appl. Genet. 133, 2363–2375. 10.1007/s00122-020-03604-1 32436020

[B30] Lu H.-J. Fellers J. P. Friesen T. L. Meinhardt S. W. Faris J. D. (2006). Genomic analysis and marker development for the *Tsn1* locus in wheat using bin-mapped ESTs and flanking BAC contigs. Theor. Appl. Genet. 112, 1132–1142. 10.1007/s00122-006-0215-4 16456656

[B31] Oerke E. C. (2006). Crop losses to pests. J. Agric. Sci. 144 (1), 31–43. 10.1017/S0021859605005708

[B32] Oraguzie N. C. Gardiner S. E. Rikkerink E. H. A. Silva H. N. (2007). Association mapping in plants (Berlin: Springer). 10.1007/978-0-387-36011-9

[B33] Patel J. S. Mamidi S. Bonman J. M. Adhikari T. B. (2013). Identification of QTL in spring wheat associated with resistance to a novel isolate of *Pyrenophora tritici-repentis* . Crop Sci. 53, 842–852. 10.2135/cropsci2012.01.0036

[B34] Patterson H. D. Thompson R. (1971). Recovery of inter-block information when block sizes are unequal. Biometrika 58 (3), 545–554. 10.1093/biomet/58.3.545

[B35] Pritchard J. K. Stephens M. Donnelly P. (2000). Inference of population structure using multilocus genotype data. Genetics 155, 945–959. 10835412 10.1093/genetics/155.2.945 PMC1461096

[B36] Rees R. G. Platz G. J. (1992). “Tan spot and its control - some Australian experiences,” in Advances in tan spot research. Eds. Francl L. J. Krupinsky J. M. McMullen M. P. (Fargo, ND, US: NDSU Agric. Exp. Sta. Publ. 146 pp), 1–15.

[B37] Riedelsheimer C. Lisec J. Czedik-Eysenberg A. Sulpice R. Flis A. Grieder C. (2012). Genome-wide association mapping of leaf metabolic profiles for dissecting complex traits in maize. Proc. Natl. Acad. Sci. USA. 109, 8872–8877. 10.1073/pnas.1120813109 22615396 PMC3384205

[B38] Sharma I. Tyagi B. S. Singh G. Venkatesh K. Gupta O. P. (2015). Enhancing wheat production- A global perspective. Indian J. Agric. Sci. 85 (1), 3–13.

[B39] Singh P. K. Gonzalez-Hernandez J. M. Goodwin S. B. (2006). Identification and molecular mapping of a gene conferring resistance to *Pyrenophora tritici-repentis* race 3 in tetraploid wheat. Phytopathology 96, 885–889. 10.1094/PHYTO-96-0885 18943754

[B40] Singh S. Bockus W. W. Sharma I. Bowden R. L. (2008). A novel source of resistance in wheat to *Pyrenophora tritici-repentis* race 1. Plant Dis. 92, 91–95. 10.1094/PDIS-92-1-0091 30786378

[B41] Singh P. K. Singh R. P. Duveiller E. Mergoum M. Adhikari T. B. Elias E. M. (2009). Genetics of wheat-*Pyrenophora tritici-repentis* interactions. Euphytica 171, 1–13. 10.1007/s10681-009-0074-6

[B42] Singh P. K. Duveiller E. Singh R. P. (2011). Evaluation of CIMMYT germplasm for resistance to leaf spotting diseases of wheat. Czech J. Genet. Plant Breed. 47, S102–S108. 10.17221/3263-CJGPB

[B43] Singh P. K. Crossa J. Duveiller E. Singh R. P. Djurle A. (2016). Association mapping for resistance to tan spot induced by *Pyrenophora tritici-repentis* race 1 in CIMMYTs historical bread wheat set. Euphytica 207, 515–525. 10.1007/s10681-015-1528-7

[B44] Singh P. K. He X. Sansaloni C. Juliana P. Dreisigacker S. Duveiller E. (2018). Resistance to spot blotch in two mapping populations of common wheat is controlled by multiple QTL of minor effects. Int. J. Mol. Sci. 19, 4054. 10.3390/ijms19124054 PMC6321084 30558200

[B45] Singh P. K. Singh S. Deng Z. He X. Kehel Z. Singh R. P. (2019). Characterization of QTLs for seedling resistance to tan spot and Septoria nodorum blotch in the PBW343/Kenya Nyangumi wheat recombinant inbred lines population. Int. J. Mol. Sci. 20, 5432. 10.3390/ijms20215432 PMC6862150 31683619

[B46] Tekauz A. (1976). Distribution, severity and relative importance of leaf spot disease of wheat in western Canada in 1974. Can. Plant Dis. Surv. 56, 36–40.

[B47] Van Raden P. M. (2008). Efficient methods to compute genomic predictions. J. Dairy Sci. 91, 4414–4423. 10.3168/jds.2007-0980 18946147

[B48] Wegulo S. N. (2011). Tan spot of cereals. The Plant Health Instructor. 10.1094/PHI-I-2011-0426-01

[B49] Zwart R. S. Thompson J. P. Milgate A. W. Bansal U. K. Williamson P. M. Raman H. (2010). QTL mapping of multiple foliar disease and root-lesion nematode resistances in wheat. Mol. Breed. 26, 107–124. 10.1007/s11032-009-9381-9

