# Supplementary figures and images for: Association Mapping of Seedling Resistance to Tan Spot (Pyrenophora tritici-repentis Race 1) in CIMMYT and South Asian Wheat Germplasm
Source: Front Plant Sci. 2020 Aug 28;11:1309. doi: 10.3389/fpls.2020.01309 (PMC7483578; doi:10.3389/fpls.2020.01309)

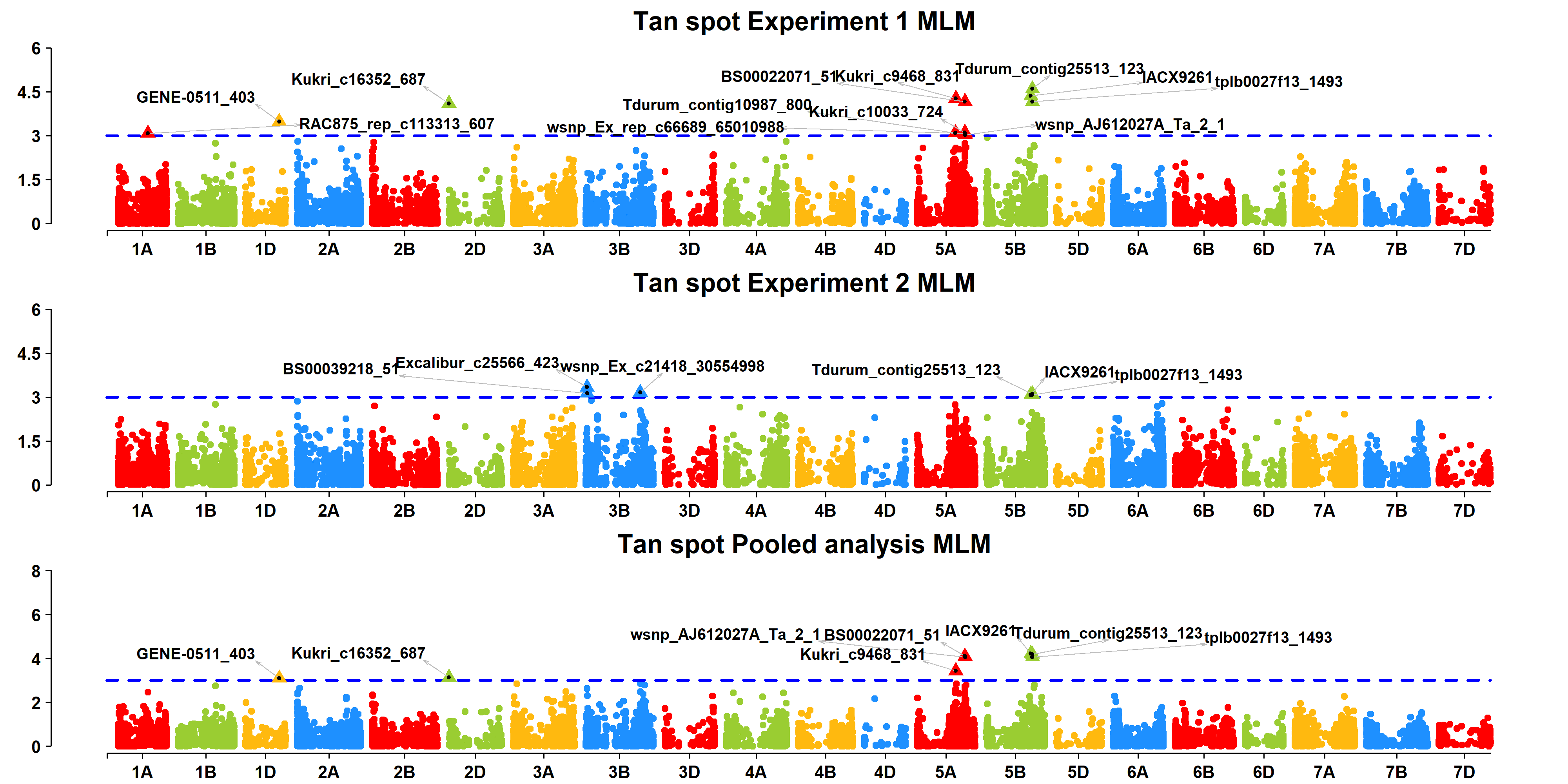

Supplement: Supplementary Figure 1 — Manhattan plots based on MLM model indicating associated markers and chromosome in experiment 1, experiment 2, and pooled analysis at LOD score 3. Foot note: X axis—chromosomes, Y axis—LOD score. [file Image_1.tiff]

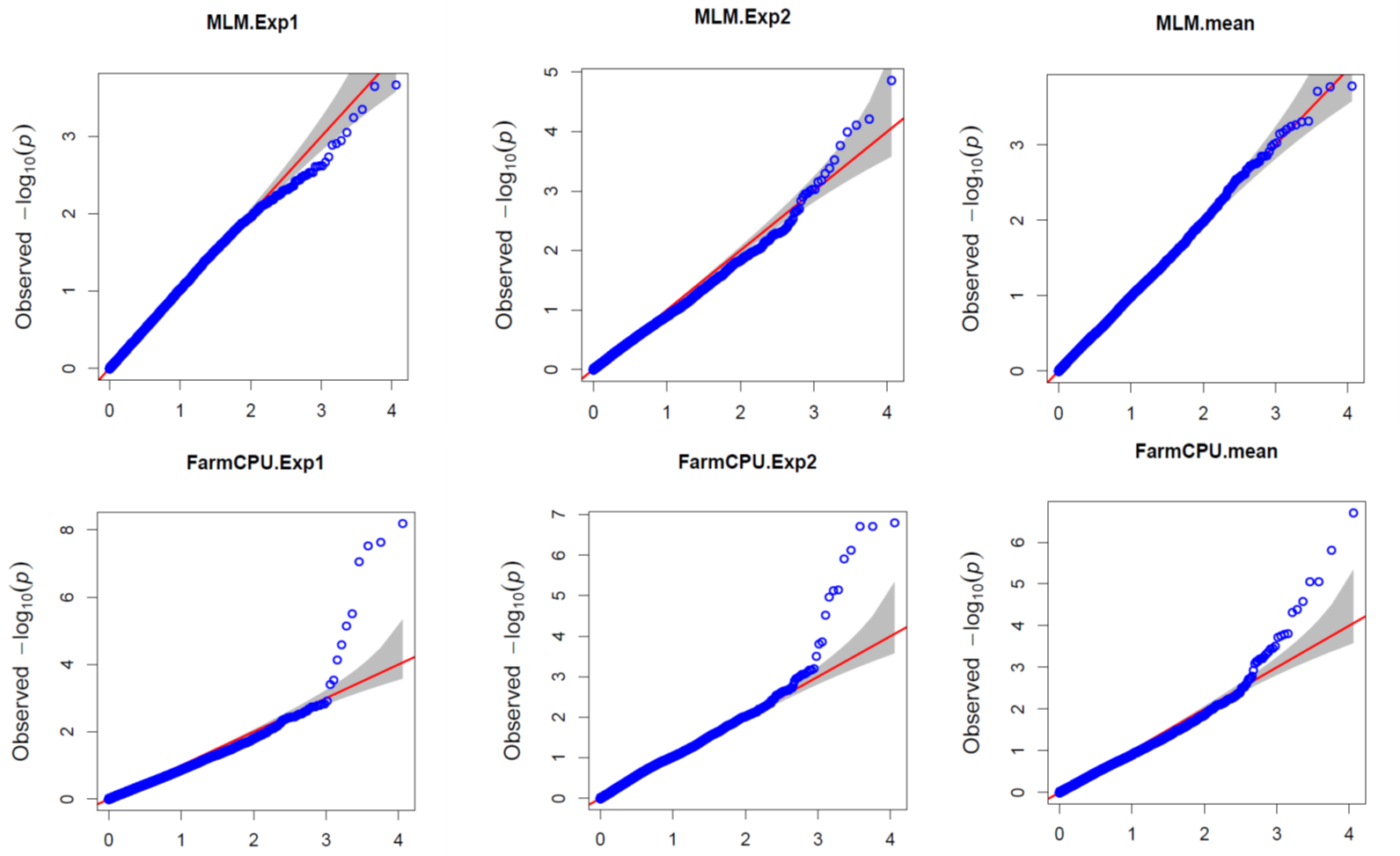

Supplement: Supplementary Figure 2 — Q–Q plot of observed vs expected log10 P values for MLM and FarmCPU GWAS models using three data sets. [file Image_2.tif]
