# Supplementary material for: Association Mapping of Seedling Resistance to Tan Spot (Pyrenophora tritici-repentis Race 1) in CIMMYT and South Asian Wheat Germplasm
Source: Front Plant Sci. 2020 Aug 28;11:1309. doi: 10.3389/fpls.2020.01309 (PMC7483578; doi:10.3389/fpls.2020.01309)
Supplement: Supplementary Table 1 — Pedigree details of 184 spring wheat genotypes used for GWAS of Tan spot [file Table_1.docx]

**Supplementary Table 1**: Pedigree details of 184 spring wheat genotypes used for GWAS of Tan spot

| **Geno ID** | **pedigree** | **Origin** |
| --- | --- | --- |
| CIM-1 | QUAIU #1/SUP152* | Mexico |
| CIM-2 | KIRITATI//HUW234+LR34/PRINIA/3/BAJ #1 | Mexico |
| CIM-3 | KIRITATI//HUW234+LR34/PRINIA/3/CHONTE/5/PRL/2*PASTOR/4/CHOIX/STAR/3/HE1/3*CNO79//2*SERI | Mexico |
| CIM-4 | CROC_1/AE.SQUARROSA (213)//PGO/10/ATTILA*2/9/KT/BAGE//FN/U/3/BZA/4/TRM/5/ALDAN/6/SERI/7/VEE#10/8/OPATA/11/ATTILA*2/PBW65 | Mexico |
| CIM-5 | BAJ #1*2//ND643/2*WBLL1 | Mexico |
| CIM-6 | PAURAQ/4/WHEAR/KUKUNA/3/C80.1/3*BATAVIA//2*WBLL1/5/PAURAQUE #1 | Mexico |
| CIM-7 | KACHU/BECARD//WBLL1*2/BRAMBLING | Mexico |
| CIM-8 | MUTUS//KIRITATI/2*TRCH/3/WHEAR/KRONSTAD F2004 | Mexico |
| CIM-9 | BAJ #1*2/TINKIO #1 | Mexico |
| CIM-10 | CROSBILL #1/DANPHE/7/CNDO/R143//ENTE/MEXI_2/3/AEGILOPS SQUARROSA (TAUS)/4/WEAVER/5/2*KAUZ/6/PRL/2*PASTOR | Mexico |
| CIM-11 | PAURAQ/5/KIRITATI/4/2*SERI.1B*2/3/KAUZ*2/BOW//KAUZ/6/PAURAQUE #1 | Mexico |
| CIM-12 | SOKOLL/3/PASTOR//HXL7573/2*BAU/5/CROC_1/AE.SQUARROSA (205)//BORL95/3/PRL/SARA//TSI/VEE#5/4/FRET2 | Mexico |
| CIM-13 | KENYA SUNBIRD/2*KACHU | Mexico |
| CIM-14 | KIRITATI//HUW234+LR34/PRINIA/3/FRANCOLIN #1/4/BAJ #1 | Mexico |
| CIM-15 | TOB/ERA//TOB/CNO67/3/PLO/4/VEE#5/5/KAUZ/6/FRET2/7/VORB/8/MILAN/KAUZ//DHARWAR DRY/3/BAV92 | Mexico |
| CIM-16 | KIRITATI//2*PRL/2*PASTOR/3/CHONTE/5/PRL/2*PASTOR/4/CHOIX/STAR/3/HE1/3*CNO79//2*SERI | Mexico |
| CIM-17 | FRET2*2/BRAMBLING//KIRITATI/2*TRCH/3/FRET2/TUKURU//FRET2 | Mexico |
| CIM-18 | KIRITATI//HUW234+LR34/PRINIA/3/CHONTE/5/PRL/2*PASTOR/4/CHOIX/STAR/3/HE1/3*CNO79//2*SERI* | Mexico |
| CIM-19 | SUP152/FRNCLN | Mexico |
| CIM-20 | BAVIS/3/ATTILA/BAV92//PASTOR/5/CROC_1/AE.SQUARROSA (205)//BORL95/3/PRL/SARA//TSI/VEE#5/4/FRET2 | Mexico |
| CIM-21 | ND643/2*WBLL1//VILLA JUAREZ F2009 | Mexico |
| CIM-22 | QUAIU #1 | Mexico |
| CIM-23 | FRANCOLIN #1/CHONTE//FRNCLN | Mexico |
| CIM-24 | TAM200/PASTOR//TOBA97/3/FRNCLN/4/WHEAR//2*PRL/2*PASTOR | Mexico |
| CIM-25 | FRANCOLIN #1* | Mexico |
| CIM-26 | VEE/MJI//2*TUI/3/PASTOR/4/BERKUT/5/BAVIS | Mexico |
| CIM-27 | DANPHE/PAURAQUE #1//MUNAL #1 | Mexico |
| CIM-28 | BAJ #1*2/KISKADEE #1 | Mexico |
| CIM-29 | SHATABDI | Mexico |
| CIM-30 | BIJOY | Mexico |
| CIM-31 | ATTILA/BAV92//PASTOR/3/ATTILA*2/PBW65 | Mexico |
| CIM-32 | WHEAR/KUKUNA/3/C80.1/3*BATAVIA//2*WBLL1/4/T.DICOCCON PI94625/AE.SQUARROSA (372)//SHA4/CHIL/5/WHEAR/KUKUNA/3/C80.1/3*BATAVIA//2*WBLL1 | Mexico |
| CIM-33 | TEPOCA T 89 | Mexico |
| CIM-34 | MILAN | Mexico |
| CIM-35 | BORLAUG100 F2014 | Mexico |
| CIM-36 | ROELFS F2007 | Mexico |
| CIM-37 | SUP152/BAJ #1 | Mexico |
| CIM-38 | ATTILA | Mexico |
| CIM-39 | VOROBEY | Mexico |
| CIM-40 | MILAN/MUNIA | Mexico |
| CIM-41 | FRANCOLIN #1 | Mexico |
| CIM-42 | SUPER 152 | Mexico |
| CIM-43 | KACHU #1 | Mexico |
| CIM-44 | MUTUS #1 | Mexico |
| CIM-45 | ATTILA*2/PBW65//WBLL1*2/TUKURU | Mexico |
| CIM-46 | BECARD/KACHU | Mexico |
| CIM-47 | BAJ #1 | Mexico |
| CIM-48 | FRNCLN/ROLF07 | Mexico |
| CIM-49 | NAC/TH.AC//3*PVN/3/MIRLO/BUC/4/2*PASTOR/5/KACHU/6/KACHU | Mexico |
| CIM-50 | FRNCLN*2/TECUE #1 | Mexico |
| CIM-51 | MUTUS*2/AKURI | Mexico |
| CIM-52 | KACHU/BECARD//WBLL1*2/BRAMBLING* | Mexico |
| CIM-53 | KAUZ/PASTOR//PBW343/3/KIRITATI/4/FRNCLN | Mexico |
| CIM-54 | QUAIU #1/SUP152 | Mexico |
| CIM-55 | SUP152/FRNCLN* | Mexico |
| CIM-56 | FRNCLN/3/ND643//2*PRL/2*PASTOR/4/FRANCOLIN #1 | Mexico |
| CIM-57 | SWSR22T.B./2*BLOUK #1//WBLL1*2/KURUKU | Mexico |
| CIM-58 | FRANCOLIN #1*2//ND643/2*WBLL1 | Mexico |
| CIM-59 | DANPHE/2*BAJ #1 | Mexico |
| CIM-60 | BAJ #1*2/BECARD | Mexico |
| CIM-61 | BAJ #1*2/5/SW89.5277/BORL95//SKAUZ/3/PRL/2*PASTOR/4/HEILO | Mexico |
| CIM-62 | FRANCOLIN #1/BAJ #1 | Mexico |
| CIM-63 | BAJ #1/3/TRCH/SRTU//KACHU | Mexico |
| CIM-64 | PBW343*2/KUKUNA//PBW343*2/KUKUNA/3/2*BAJ #1 | Mexico |
| CIM-65 | BAJ #1/5/ATTILA/3*BCN//BAV92/3/TILHI/4/SHA7/VEE#5//ARIV92 | Mexico |
| CIM-66 | BAJ #1/3/TRCH/SRTU//KACHU | Mexico |
| CIM-67 | SUP152/3/INQALAB 91*2/TUKURU//WHEAR | Mexico |
| CIM-68 | SUP152/3/TRCH/SRTU//KACHU | Mexico |
| CIM-69 | ATTILA*2/PBW65//KACHU/3/UP2338*2/KKTS*2//YANAC | Mexico |
| CIM-70 | KSW/SAUAL//SAUAL/3/BORL14 | Mexico |
| CIM-71 | KFA/2*KACHU/3/ATTILA*2/PBW65//MURGA | Mexico |
| CIM-72 | ATTILA*2/PBW65*2//KACHU/3/TRCH/HUIRIVIS #1 | Mexico |
| CIM-73 | BORL14//KFA/2*KACHU-1 | Mexico |
| CIM-74 | BORL14//KFA/2*KACHU-2 | Mexico |
| CIM-75 | BORL14//KFA/2*KACHU-3 | Mexico |
| CIM-76 | BORL14//KFA/2*KACHU-4 | Mexico |
| CIM-77 | KACHU/KIRITATI//BORL14 | Mexico |
| CIM-78 | KSW/SAUAL//SAUAL/3/BORL14-1 | Mexico |
| CIM-79 | KSW/SAUAL//SAUAL/3/BORL14-2 | Mexico |
| CIM-80 | KSW/SAUAL//SAUAL/3/BORL14-3 | Mexico |
| CIM-81 | KSW/SAUAL//SAUAL/3/BORL14-4 | Mexico |
| CIM-82 | ORL93320/ER2000 | Mexico |
| CIM-83 | SW91.4903/3/URES/BOW//OPATA/4/SW94.15373 | Mexico |
| CIM-84 | MON/TAN//ROMO96/3/METSO/4/FINSI | Mexico |
| CIM-85 | MUU | Mexico |
| CIM-86 | PFAU/WEAVER*2/3/WEAVER/ESDA//BORL95 | Mexico |
| CIM-87 | SW8488*2/KURUKU | Mexico |
| CIM-88 | FINSI/METSO-1 | Mexico |
| CIM-89 | FINSI/METSO-2 | Mexico |
| CIM-90 | FINSI/METSO-3 | Mexico |
| CIM-91 | BAV92//IRENA/KAUZ/3/HUITES | Mexico |
| CIM-92 | GONDO//BAU/MILAN/3/PASTOR | Mexico |
| CIM-93 | ATTILA/3*BCN//BAV92/3/TILHI | Mexico |
| CIM-94 | KACHU #1-1 | Mexico |
| CIM-95 | TEG/MIAN YANG 20//CHUM18/5*BCN | Mexico |
| CIM-96 | JWS 17/7/IAS58/4/KAL/BB//CJ71/3/ALD/5/CNR/6/THB/CEP7780/8/FINSI | Mexico |
| CIM-97 | MILAN/ARA90//TNMU/TUI | Mexico |
| BGD-1 | PRODIP | Bangladesh |
| BGD-2 | BARI GOM 25 | Bangladesh |
| BGD-3 | BARI GOM 26 | Bangladesh |
| BGD-4 | BARI GOM 28 | Bangladesh |
| BGD-5 | BARI GOM 29 | Bangladesh |
| BGD-6 | BARI GOM 30 | Bangladesh |
| BGD-7 | BAW-1182 | Bangladesh |
| BGD-8 | BAW-1194 | Bangladesh |
| BGD-9 | BAW-1195 | Bangladesh |
| BGD-10 | BAW-1200 | Bangladesh |
| BGD-11 | BAW-1202 | Bangladesh |
| BGD-12 | BAW-1203 | Bangladesh |
| BGD-13 | BAW-1208 | Bangladesh |
| BGD-14 | BAW-1209 | Bangladesh |
| BGD-15 | BAW-1219 | Bangladesh |
| BGD-16 | BAW-1222 | Bangladesh |
| BGD-17 | BAW-1249 | Bangladesh |
| BGD-18 | BAW-1260 | Bangladesh |
| BGD-19 | SOKOLL/ROLF07 | Bangladesh |
| IND-1 | HD 2967 | India |
| IND-2 | DBW 90 | India |
| IND-3 | HD 3043 | India |
| IND-4 | DBW 88 | India |
| IND-5 | HD 3059 | India |
| IND-6 | HD 3086 | India |
| IND-7 | PBW 644 | India |
| IND-8 | WH 1021 | India |
| IND-9 | WH 1080 | India |
| IND-10 | WH 1105 | India |
| IND-11 | WH 1124 | India |
| IND-12 | WH 1142 | India |
| IND-13 | HD 3171 | India |
| IND-14 | K 1317 | India |
| IND-15 | C 306 | India |
| IND-16 | DBW 39 | India |
| IND-17 | HD2733 | India |
| IND-18 | HD 2888 | India |
| IND-19 | MAGHAR | India |
| IND-20 | K 0307 | India |
| IND-21 | K1006 | India |
| IND-22 | HI 8759 | India |
| IND-23 | HD 4728 | India |
| IND-24 | HI 8498 | India |
| IND-25 | HI 8737 | India |
| IND-26 | MPO 1215 | India |
| IND-27 | MACS 3949 | India |
| IND-28 | HI 1605 | India |
| IND-29 | AKDW 2997-16 | India |
| IND-30 | DBW 93 | India |
| IND-31 | HD 2932 | India |
| IND-32 | MACS 6222 | India |
| IND-33 | MACS 6478 | India |
| IND-34 | NI 5439 | India |
| IND-35 | NIAW 1415 | India |
| IND-36 | NIAW 34 | India |
| IND-37 | NIDW 295 | India |
| IND-38 | RAJ 4083 | India |
| IND-39 | UAS 428 | India |
| IND-40 | UAS 446 | India |
| NPL-1 | BHRIKUTI | Nepal |
| NPL-2 | GAUTAM | Nepal |
| NPL-3 | WK1204 | Nepal |
| NPL-4 | ADITYA | Nepal |
| NPL-5 | VIJAY | Nepal |
| NPL-6 | DHAWALAGIRI | Nepal |
| NPL-7 | BL 3623 | Nepal |
| NPL-8 | BL3629 | Nepal |
| NPL-9 | BL4341 | Nepal |
| NPL-10 | BL4407 | Nepal |
| NPL-11 | BL4725 | Nepal |
| NPL-12 | BL4707 | Nepal |
| NPL-13 | BL 4406 | Nepal |
| NPL-14 | BL4682 | Nepal |
| NPL-15 | BL4621 | Nepal |
| NPL-16 | BL4622 | Nepal |
| NPL-17 | NL1164 | Nepal |
| NPL-18 | NL1202 | Nepal |
| NPL-19 | NL1249 | Nepal |
| NPL-20 | NL1270 | Nepal |
| NPL-21 | NL1178 | Nepal |
| NPL-22 | NL1226 | Nepal |
| NPL-23 | NL1231 | Nepal |
| NPL-24 | WK2432 | Nepal |
| NPL-25 | WK2375 | Nepal |
| NPL-26 | WK2123 | Nepal |
| NPL-27 | WK2286 | Nepal |
| NPL-28 | WK2395 | Nepal |
