# Supplementary material for: Association Mapping of Seedling Resistance to Tan Spot (Pyrenophora tritici-repentis Race 1) in CIMMYT and South Asian Wheat Germplasm
Source: Front Plant Sci. 2020 Aug 28;11:1309. doi: 10.3389/fpls.2020.01309 (PMC7483578; doi:10.3389/fpls.2020.01309)
Supplement: Supplementary Table 2 — Presence and absence scoring of resistant allele for identifying nine MTAs in 184 wheat genotypes [file Table_2.docx]

**Supplementary Table 2:** Presence and absence scoring of resistant allele for identify nine MTAs in 184 wheat genotypes

| **Geno-ID** | **BobWhite_c28635_785** | **Excalibur_c34937_710** | **AX-94880001** | **wsnp_Ex_c4063_7344449** | **wsnp_Ex_c12450_19850925** | **TA001138-0446** | **TA001138-0446** | **IACX9261** | **RAC875_c103443_475** | **Kukri_c15768_1383** | **Tan spot score** | | |
| --- | --- | --- | --- | --- | --- | --- | --- | --- | --- | --- | --- | --- | --- |
|  | **1B** | **2A** | **2B** | **3B** | **4A** | **5A** | **5A** | **5B** | **6A** | **7D** | **Exp 1** | **Exp 2** | **Pooled** |
| Erik |  |  |  |  |  |  |  |  |  |  | 1.0 | 1.1 | 1.0 |
| Glenlea |  |  |  |  |  |  |  |  |  |  | 4.9 | 4.3 | 4.6 |
| 6B- 365 |  |  |  |  |  |  |  |  |  |  | 2.1 | 3.0 | 2.6 |
| 6B- 662 |  |  |  |  |  |  |  |  |  |  | 2.4 | 2.3 | 2.3 |
| CIM-1 | + | - | + | - | - | - | - | + | + | - | 1.4 | 2.2 | 1.8 |
| CIM-2 | - | - | + | - | - | - | - | + | - | - | 1.3 | 1.8 | 1.6 |
| CIM-3 | + | - | + | - | - | - | - | + | + | - | 1.0 | 1.3 | 1.2 |
| CIM-4 | + | - | + | + | - | - | - | + | - | - | 1.6 | 2.1 | 1.9 |
| CIM-5 | - | - | + | - | + | - | - | + | - | - | 2.7 | 2.3 | 2.5 |
| CIM-6 | + | - | + | - | - | - | - | + | - | - | 1.5 | 1.9 | 1.7 |
| CIM-7 | - | - | + | - | - | - | - | + | - | - | 3.0 | 2.4 | 2.7 |
| CIM-8 | - | - | + | - | - | - | - | + | + | - | 2.3 | 2.0 | 2.2 |
| CIM-9 | - | - | + | - | + | - | - | + | - | - | 2.3 | 2.5 | 2.4 |
| CIM-10 | + | - | + | - | - | - | - | - | - | - | 2.1 | 2.7 | 2.4 |
| CIM-11 | + | - | + | - | - | - | - | + | - | + | 1.5 | 1.5 | 1.5 |
| CIM-12 | - | + | + | - | - | - | - | + | + | - | 1.8 | 2.1 | 2.0 |
| CIM-13 | - | - | + | + | - | - | - | + | + | - | 2.3 | 2.6 | 2.5 |
| CIM-14 | - | - | + | - | - | - | - | + | + | - | 1.8 | 2.4 | 2.1 |
| CIM-15 | - | - | + | - | - | - | - | - | + | - | 2.3 | 1.8 | 2.1 |
| CIM-16 | + | - | + | - | - | - | - | - | + | - | 3.0 | 3.2 | 3.1 |
| CIM-17 | + | - | + | - | - | - | - | - | - | - | 2.5 | 2.6 | 2.6 |
| CIM-18 | + | - | + | - | + | - | - | - | + | + | 4.3 | 3.2 | 3.8 |
| CIM-19 | + | - | + | - | - | - | - | + | - | - | 1.7 | 1.8 | 1.8 |
| CIM-20 | - | - | + | - | - | - | - | + | - | + | 2.5 | 2.3 | 2.4 |
| CIM-21 | - | - | - | - | - | - | - | + | - | + | 2.1 | 2.5 | 2.3 |
| CIM-22 | - | - | + | - | - | - | - | + | + | - | 1.7 | 2.4 | 2.1 |
| CIM-23 | + | - | + | - | - | - | - | + | + | - | 1.3 | 1.8 | 1.6 |
| CIM-24 | + | - | + | - | - | - | - | + | - | + | 1.7 | 2.1 | 1.9 |
| CIM-25 | + | - | + | - | - | - | - | + | + | - | 1.4 | 1.3 | 1.4 |
| CIM-26 | + | - | + | - | - | - | - | + | + | + | 1.3 | 1.3 | 1.3 |
| CIM-27 | + | - | + | - | + | - | - | - | - | - | 1.7 | 2.0 | 1.9 |
| CIM-28 | + | - | - | - | + | - | - | + | - | - | 1.4 | 2.0 | 1.7 |
| CIM-29 | + | - | + | - | - | - | - | + | + | - | 1.6 | 2.1 | 1.9 |
| CIM-30 | + | + | + | - | + | + | + | + | + | - | 2.9 | 3.3 | 3.1 |
| CIM-31 | - | - | + | + | - | - | - | + | - | - | 2.0 | 2.4 | 2.2 |
| CIM-32 | + | - | + | - | - | - | - | + | - | - | 1.2 | 1.3 | 1.3 |
| CIM-33 | + | - | + | - | - | + | + | + | + | - | 1.7 | 2.7 | 2.2 |
| CIM-34 | + | + | + | + | - | - | + | + | - | - | 1.5 | 2.3 | 1.9 |
| CIM-35 | - | - | + | - | - | - | - | + | - | - | 1.0 | 1.7 | 1.4 |
| CIM-36 | + | - | + | - | + | - | - | + | - | - | 1.6 | 2.3 | 2.0 |
| CIM-37 | + | - | + | - | + | - | - | + | - | - | 1.6 | 1.8 | 1.7 |
| CIM-38 | + | - | + | - | - | - | - | + | - | - | 1.0 | 1.3 | 1.2 |
| CIM-39 | + | - | + | - | + | - | - | + | + | + | 1.3 | 2.1 | 1.7 |
| CIM-40 | + | - | + | - | - | - | - | - | + | - | 1.3 | 2.7 | 2.0 |
| CIM-41 | + | - | + | - | - | - | - | + | - | - | 1.0 | 1.2 | 1.1 |
| CIM-42 | + | - | + | - | - | - | - | + | - | - | 1.4 | 1.7 | 1.6 |
| CIM-43 | - | - | + | - | - | - | - | + | + | - | 1.6 | 2.6 | 2.1 |
| CIM-44 | - | - | + | - | - | - | - | + | - | - | 1.2 | 1.6 | 1.4 |
| CIM-45 | - | - | + | - | - | - | - | + | - | - | 1.3 | 2.4 | 1.9 |
| CIM-46 | - | - | + | - | - | - | - | + | - | - | 1.4 | 1.9 | 1.7 |
| CIM-47 | + | - | + | - | + | - | - | + | - | - | 1.5 | 2.3 | 1.9 |
| CIM-48 | + | - | + | - | + | - | - | + | - | - | 1.8 | 2.3 | 2.1 |
| CIM-49 | + | - | + | - | - | - | - | + | + | - | 1.0 | 1.9 | 1.5 |
| CIM-50 | + | - | + | - | - | - | - | + | - | - | 1.0 | 1.1 | 1.1 |
| CIM-51 | - | - | + | + | - | - | - | + | - | - | 1.3 | 1.5 | 1.4 |
| CIM-52 | - | - | + | - | - | - | - | + | - | - | 1.7 | 2.0 | 1.9 |
| CIM-53 | + | - | + | - | - | - | - | + | - | - | 1.3 | 1.3 | 1.3 |
| CIM-54 | + | - | + | - | - | - | - | + | + | - | 1.3 | 1.8 | 1.6 |
| CIM-55 | + | - | + | - | - | - | - | + | - | - | 1.2 | 1.2 | 1.2 |
| CIM-56 | - | - | + | - | - | - | - | + | - | - | 1.5 | 2.6 | 2.1 |
| CIM-57 | - | - | - | - | - | - | - | + | - | - | 1.3 | 1.1 | 1.2 |
| CIM-58 | + | - | - | - | - | - | - | + | - | - | 1.3 | 1.8 | 1.6 |
| CIM-59 | + | - | + | - | + | - | - | + | - | - | 1.2 | 1.9 | 1.6 |
| CIM-60 | + | - | + | - | - | - | - | + | - | - | 1.9 | 1.7 | 1.8 |
| CIM-61 | + | - | + | - | - | - | - | + | + | + | 2.8 | 1.5 | 2.2 |
| CIM-62 | + | - | + | - | - | - | - | + | - | - | 1.3 | 1.6 | 1.5 |
| CIM-63 | - | - | + | - | - | - | - | + | - | - | 1.1 | 1.0 | 1.1 |
| CIM-64 | + | - | + | - | + | - | - | + | - | - | 1.4 | 1.7 | 1.6 |
| CIM-65 | + | - | + | - | - | - | - | + | - | - | 1.5 | 1.6 | 1.6 |
| CIM-66 | - | - | + | - | + | - | - | + | - | - | 1.5 | 2.0 | 1.8 |
| CIM-67 | + | - | + | - | - | - | - | + | - | - | 1.3 | 1.3 | 1.3 |
| CIM-68 | + | - | + | - | - | - | - | + | - | - | 1.4 | 1.3 | 1.4 |
| CIM-69 | + | - | + | - | - | - | - | + | + | - | 1.5 | 1.7 | 1.6 |
| CIM-70 | - | - | + | - | - | + | + | + | - | - | 2.6 | 1.8 | 2.2 |
| CIM-71 | - | - | + | - | - | - | - | + | + | - | 2.3 | 1.7 | 2.0 |
| CIM-72 | + | - | + | - | - | - | - | + | + | - | 1.4 | 1.3 | 1.4 |
| CIM-73 | - | - | + | - | - | - | - | + | + | - | 1.8 | 2.2 | 2.0 |
| CIM-74 | - | - | + | - | - | - | - | + | + | - | 1.3 | 2.1 | 1.7 |
| CIM-75 | - | - | + | - | - | - | - | + | + | - | 1.0 | 1.5 | 1.3 |
| CIM-76 | - | - | + | - | - | - | - | + | + | - | 1.6 | 1.5 | 1.6 |
| CIM-77 | - | - | + | - | - | - | - | + | + | - | 1.1 | 1.1 | 1.1 |
| CIM-78 | - | - | + | - | - | + | + | - | + | - | 3.3 | 2.5 | 2.9 |
| CIM-79 | - | - | + | - | - | + | + | - | + | - | 2.6 | 2.7 | 2.7 |
| CIM-80 | - | - | + | - | - | + | + | - | + | - | 2.7 | 2.3 | 2.5 |
| CIM-81 | - | - | + | - | - | - | - | + | + | - | 1.3 | 1.9 | 1.6 |
| CIM-82 | + | - | + | - | + | - | - | + | + | - | 1.4 | 2.0 | 1.7 |
| CIM-83 | + | - | + | - | + | - | - | + | - | + | 1.3 | 1.3 | 1.3 |
| CIM-84 | + | - | + | - | + | - | - | - | + | + | 2.2 | 2.3 | 2.3 |
| CIM-85 | + | - | + | - | + | - | - | + | + | - | 1.8 | 2.3 | 2.1 |
| CIM-86 | + | - | + | - | - | - | - | + | + | - | 1.5 | 2.0 | 1.8 |
| CIM-87 | + | - | + | - | - | - | - | + | + | + | 1.1 | 1.1 | 1.1 |
| CIM-88 | + | + | + | - | - | - | - | - | + | + | 2.0 | 2.8 | 2.4 |
| CIM-89 | + | + | + | - | - | - | - | - | + | + | 2.5 | 3.4 | 3.0 |
| CIM-90 | + | + | + | - | - | - | - | - | + | + | 2.8 | 2.9 | 2.9 |
| CIM-91 | - | - | + | - | - | - | - | + | + | - | 1.8 | 2.2 | 2.0 |
| CIM-92 | + | - | + | - | - | - | - | - | + | - | 2.1 | 2.5 | 2.3 |
| CIM-93 | + | - | + | - | - | - | - | - | - | - | 1.4 | 1.3 | 1.4 |
| CIM-94 | - | - | + | - | - | - | - | + | + | - | 1.3 | 1.9 | 1.6 |
| CIM-95 | + | - | + | - | + | - | - | + | + | - | 1.2 | 1.3 | 1.3 |
| CIM-96 | + | - | + | - | + | + | + | - | + | - | 1.8 | 2.3 | 2.1 |
| CIM-97 | + | + | + | - | - | + | + | + | - | - | 1.7 | 2.3 | 2.0 |
| BGD-1 | + | + | - | - | - | + | + | + | - | - | 1.8 | 2.9 | 2.4 |
| BGD-2 | + | - | - | - | + | + | - | + | - | - | 1.6 | 2.0 | 1.8 |
| BGD-3 | + | - | + | + | - | - | - | + | + | - | 2.3 | 2.6 | 2.5 |
| BGD-4 | + | - | + | - | + | - | - | + | + | - | 2.2 | 2.3 | 2.3 |
| BGD-5 | - | - | + | - | - | - | - | + | - | - | 1.0 | 1.1 | 1.1 |
| BGD-6 | + | - | + | + | - | - | - | + | - | - | 1.7 | 2.4 | 2.1 |
| BGD-7 | + | - | + | - | + | - | - | + | - | - | 1.4 | 1.9 | 1.7 |
| BGD-8 | + | - | - | - | - | - | - | + | + | - | 1.5 | 1.7 | 1.6 |
| BGD-9 | + | - | - | + | - | + | + | + | - | - | 1.6 | 1.2 | 1.4 |
| BGD-10 | + | - | + | - | - | - | - | + | - | - | 3.3 | 1.7 | 2.5 |
| BGD-11 | + | - | + | - | - | + | + | + | + | + | 2.9 | 2.8 | 2.9 |
| BGD-12 | + | - | + | - | - | + | + | + | + | + | 2.5 | 3.3 | 2.9 |
| BGD-13 | + | + | + | - | - | + | + | + | + | - | 2.5 | 2.6 | 2.6 |
| BGD-14 | + | - | - | - | + | - | - | + | - | - | 2.6 | 2.1 | 2.4 |
| BGD-15 | + | - | - | - | + | + | + | + | + | - | 1.8 | 2.4 | 2.1 |
| BGD-16 | + | - | + | - | - | + | + | + | + | + | 2.8 | 2.6 | 2.7 |
| BGD-17 | + | - | - | - | + | + | + | + | - | - | 2.7 | 2.3 | 2.5 |
| BGD-18 | - | - | + | - | - | - | - | + | + | - | 2.1 | 1.8 | 2.0 |
| BGD-19 | + | + | + | - | - | - | - | + | - | - | 1.7 | 2.3 | 2.0 |
| IND-1 | + | - | + | - | - | - | - | + | - | - | 1.0 | 1.1 | 1.1 |
| IND-2 | + | - | + | - | - | - | - | - | + | - | 1.9 | 1.9 | 1.9 |
| IND-3 | + | - | - | - | - | - | - | + | - | - | 1.0 | 1.5 | 1.3 |
| IND-4 | - | - | + | - | - | - | - | + | + | - | 1.8 | 2.1 | 2.0 |
| IND-5 | - | - | + | - | - | - | - | + | + | - | 1.5 | 2.1 | 1.8 |
| IND-6 | + | - | + | - | - | - | - | - | + | - | 1.8 | 2.8 | 2.3 |
| IND-7 | + | - | + | - | - | - | - | + | + | - | 1.5 | 1.6 | 1.6 |
| IND-8 | - | - | + | - | + | - | - | + | - | - | 2.8 | 2.3 | 2.6 |
| IND-9 | + | - | + | - | - | - | - | - | + | + | 1.5 | 1.8 | 1.7 |
| IND-10 | - | - | + | - | - | - | - | + | - | - | 1.4 | 1.5 | 1.5 |
| IND-11 | + | - | + | - | - | - | - | - | + | - | 1.7 | 2.6 | 2.2 |
| IND-12 | + | - | + | - | - | - | - | - | - | - | 1.5 | 1.8 | 1.7 |
| IND-13 | + | - | + | - | + | - | - | + | + | - | 1.5 | 1.8 | 1.7 |
| IND-14 | + | - | + | + | + | - | - | + | + | + | 1.3 | 1.3 | 1.3 |
| IND-15 | - | - | + | - | - | - | - | + | + | - | 1.5 | 2.4 | 2.0 |
| IND-16 | + | - | + | - | - | - | - | - | - | - | 1.4 | 1.5 | 1.5 |
| IND-17 | + | - | + | - | + | - | - | + | - | - | 1.0 | 1.0 | 1.0 |
| IND-18 | - | - | + | - | - | - | - | + | + | - | 2.7 | 2.0 | 2.4 |
| IND-19 | - | - | + | - | - | - | - | + | + | - | 1.4 | 2.0 | 1.7 |
| IND-20 | - | - | - | - | - | - | - | + | - | - | 1.3 | 1.6 | 1.5 |
| IND-21 | - | - | - | - | - | - | - | + | - | - | 1.0 | 1.6 | 1.3 |
| IND-22 | + | + | + | + | - | - | - | + | + | + | 1.5 | 1.7 | 1.6 |
| IND-23 | + | + | + | + | - | - | - | + | + | + | 1.3 | 1.3 | 1.3 |
| IND-24 | + | + | + | + | - | - | + | + | + | + | 1.7 | 1.8 | 1.8 |
| IND-25 | + | + | + | + | - | - | - | + | + | + | 1.8 | 1.7 | 1.8 |
| IND-26 | + | - | + | + | - | - | - | + | + | - | 1.5 | 1.1 | 1.3 |
| IND-27 | + | + | + | + | - | - | - | + | + | + | 1.0 | 1.1 | 1.1 |
| IND-28 | + | - | + | - | - | - | - | + | + | + | 1.4 | 1.4 | 1.4 |
| IND-29 | + | + | + | + | - | - | - | + | + | + | 2.3 | 2.4 | 2.4 |
| IND-30 | + | - | + | - | - | + | + | + | - | - | 1.4 | 1.2 | 1.3 |
| IND-31 | - | - | + | - | - | + | + | - | + | - | 3.3 | 2.7 | 3.0 |
| IND-32 | + | - | + | - | + | - | - | - | - | - | 3.3 | 2.6 | 3.0 |
| IND-33 | - | + | + | - | - | - | - | + | - | - | 1.0 | 1.1 | 1.1 |
| IND-34 | - | - | + | - | - | + | + | - | - | - | 2.9 | 2.3 | 2.6 |
| IND-35 | + | - | + | - | - | - | - | - | - | - | 2.6 | 2.3 | 2.5 |
| IND-36 | - | - | + | - | - | + | + | - | + | - | 2.8 | 2.6 | 2.7 |
| IND-37 | + | + | + | + | - | - | - | + | + | + | 1.3 | 1.0 | 1.2 |
| IND-38 | - | - | - | - | + | + | + | + | + | - | 2.1 | 2.8 | 2.5 |
| IND-39 | + | + | + | + | - | - | - | + | + | + | 2.0 | 2.5 | 2.3 |
| IND-40 | + | + | + | + | - | - | - | + | + | + | 1.3 | 1.8 | 1.6 |
| NPL-1 | - | - | + | - | - | - | - | + | + | - | 2.9 | 2.9 | 2.9 |
| NPL-2 | + | - | - | + | - | + | + | + | + | + | 3.2 | 3.1 | 3.2 |
| NPL-3 | + | - | - | - | - | + | + | + | - | - | 1.8 | 2.1 | 2.0 |
| NPL-4 | + | - | - | - | - | + | + | + | + | - | 2.7 | 2.1 | 2.4 |
| NPL-5 | + | - | - | - | - | - | - | + | - | - | 1.3 | 1.6 | 1.5 |
| NPL-6 | + | - | + | - | - | - | - | + | + | - | 1.3 | 1.6 | 1.5 |
| NPL-7 | + | - | - | - | - | - | - | + | - | - | 1.3 | 1.1 | 1.2 |
| NPL-8 | + | - | - | - | - | - | - | + | - | - | 1.4 | 1.4 | 1.4 |
| NPL-9 | + | - | - | - | - | - | - | + | - | - | 1.3 | 1.3 | 1.3 |
| NPL-10 | - | - | - | + | - | - | - | + | - | - | 1.0 | 1.0 | 1.0 |
| NPL-11 | + | - | - | - | - | - | - | + | - | - | 1.0 | 1.2 | 1.1 |
| NPL-12 | + | - | - | - | + | - | - | - | - | - | 2.3 | 2.4 | 2.4 |
| NPL-13 | - | - | - | + | - | - | - | + | - | - | 1.5 | 1.9 | 1.7 |
| NPL-14 | + | - | - | - | + | + | + | + | - | - | 1.8 | 1.8 | 1.8 |
| NPL-15 | - | - | - | - | + | - | - | + | - | - | 1.5 | 2.0 | 1.8 |
| NPL-16 | - | - | - | - | + | - | - | + | - | - | 1.5 | 1.8 | 1.7 |
| NPL-17 | - | - | + | + | - | - | - | + | + | - | 1.5 | 1.8 | 1.7 |
| NPL-18 | - | - | + | - | - | - | - | + | + | - | 2.1 | 2.3 | 2.2 |
| NPL-19 | + | + | + | - | - | - | - | - | + | - | 1.8 | 2.5 | 2.2 |
| NPL-20 | + | - | + | - | - | - | - | + | + | - | 1.1 | 1.8 | 1.5 |
| NPL-21 | + | + | + | - | - | - | - | - | - | - | 2.2 | 2.8 | 2.5 |
| NPL-22 | - | + | + | - | - | - | - | + | - | - | 2.7 | 2.0 | 2.4 |
| NPL-23 | + | - | + | - | - | - | - | + | - | - | 1.3 | 1.7 | 1.5 |
| NPL-24 | - | - | - | - | - | - | - | + | + | - | 1.9 | 2.3 | 2.1 |
| NPL-25 | - | - | - | - | + | + | + | + | + | - | 3.2 | 2.7 | 3.0 |
| NPL-26 | + | - | - | - | - | + | + | + | + | - | 1.6 | 1.8 | 1.7 |
| NPL-27 | + | - | - | - | - | - | - | + | - | - | 1.4 | 1.7 | 1.6 |
| NPL-28 | + | - | - | - | - | + | + | + | + | - | 1.5 | 1.3 | 1.4 |

+ Resistant allele - Susceptible allele Exp= Experiment

Erick = Resistant check, Glenlea = Susceptible check, 6B- 365 = moderately susceptible check, 6B- 662 = moderately resistant check
